# A new species of *Eigenmannia* Jordan & Evermann (Gymnotiformes: Sternopygidae) from rio Tapajós, Brazil, with discussion on its species group and the myology within Eigenmanniinae

**DOI:** 10.1371/journal.pone.0220287

**Published:** 2019-08-14

**Authors:** Luiz Antônio Wanderley Peixoto, Willian M. Ohara

**Affiliations:** 1 Museu de Zoologia da Universidade de São Paulo, Seção de peixes, São Paulo, São Paulo, Brazil; 2 Universidade Federal de Rondônia, Departamento de Engenharia de Pesca, Presidente Médici, Rondônia, Brazil; National Scientific and Technical Research Council (CONICET), ARGENTINA

## Abstract

A new species of *Eigenmannia* is described from the rio Mutum, tributary of upper rio Juruena, rio Tapajós basin, Comodoro, Mato Grosso, Brazil. The new species is distinguished from all congeners by coloration pattern, position of the mouth, number of scales rows above lateral line, number of premaxillary and dentary teeth, number of precaudal vertebrae, orbital diameter, mouth width, relative depth of posterodorsal expansion on infraorbitals 1+2 and relative size of coronomeckelian bone. Comments on potentially useful characters in phylogenetic studies derived from musculature, discussion on *Eigenmannia* species-group and the first dichotomous key for *Eigenmannia* are provided.

## Introduction

Species allocated in *Eigenmannia* Jordan & Evermann are small- to medium-sized omnivores (up to 350 mm of total length), with insectivorous trends [1–3]. They inhabit floodplains, terra firme streams, river channels or caves [4,5], and use a monophasic electric organ discharge to discharges ranging from 100 to 780 Hz in order to communicate and explore environment [6]. *Eigenmannia*, known as the “glass electric knifefish” or “ituí transparente” in the aquarium trade, is the most species-rich genus in Sternopygidae, currently with 22 valid species (besides “*Eigenmannia*” *goajira* Schultz provisionally *incertae sedis*; [7]) widely distributed from Río Tuíra basin, Panamá, to Río de La Plata basin, Argentina [8–10]. Its largest diversity is found in the Amazon basin, where approximately half of the known species occur. Despite several taxonomic contributions (e.g., [11–17]), the monophyly of *Eigenmannia* is currently doubtful (*cf*. [18–21]), and its taxonomy is grounded on group of species, a similar condition found in several Neotropical fishes that are waiting for taxonomic and phylogenetics efforts to bring light to its classification (e.g. *Apistogramma* Regan, *Corydoras* Lacepède, *Hyphessobrycon* Durbin, *Moenkhausia* Eigenmann).

Alves-Gomes [22] was the first author who proposed species group within of a phylogenetic context. The author, based on molecular evidences, presented a clade named as “*Eigenmannia virescens* group” composed by five undescribed species. A few years later, Albert [20] did not recover the *Eigenmannia* monophyly and proposed two species groups: “*Eigenmannia microstoma* species-group” and “*Eigenmannia virescens* species-group”. Due to difficulties in recognizing the characters or the taxonomic compositions considered in previous studies, Peixoto *et al*. [8] proposed “*Eigenmannia trilineata* species-group” to allocate those species that share the presence of superior midlateral stripe. This feature corresponds to a concentration of chromatophores between the lateral line and the proximal portion of the pterygiophores of the anal fin, a condition shared exclusively in this group among all Gymnotiformes. At the beginning, the *Eigenmannia trilineata* species-group was proposed to include *E*. *microstoma* (Reinhardt) (removed from *E*. *microstoma* species-group—*sensu* [20]), *E*. *trilineata* López & Castello (reallocated from *E*. *virescens* species-group—*sensu* [20]), *E*. *vicentespelaea* Triques and seven species described in that study (*E*. *antonioi* Peixoto, Dutra & Wosiacki, *E*. *desantanai* Peixoto, Dutra & Wosiacki, *E*. *guairaca* Peixoto, Dutra & Wosiacki, *E*. *matintapereira* Peixoto, Dutra & Wosiacki, *E*. *muirapinima* Peixoto, Dutra & Wosiacki, *E*. *pavulagem* Peixoto, Dutra & Wosiacki, and *E*. *waiwai* Peixoto, Dutra & Wosiacki). More recently, *E*. *besouro* Peixoto & Wosiacki, *E*. *correntes* Campos-da-Paz & Queiroz, *E*. *sayona* Peixoto & Waltz, and *E*. *loretana* Waltz & Albert were described and assigned to the *Eigenmannia trilineata* species-group, totaling 14 valid species [23–26], representing the highest diversity within the genus, and even within the remaining genera of Sternopygidae.

Recently, Waltz & Albert [26] proposed the “*Eigenmannia macrops* species-group” and “*Eigenmannia humboldtii* species-group”. Subsequently, Waltz & Albert [10] rediagnosed the *Eigenmannia humboldtii* species-group by: (1) body size larger than 30 cm of total length, (2) body depth greater than 18% of the length to the end of anal fin, (3) broad and opaque body in life, and (4) absence of longitudinal stripes.

Recent fieldwork in the upper rio Juruena, rio Tapajós basin, yielded an additional and very distinctive undescribed species related to the *Eigenmannia trilineata* species-group, which enabled us now to describe the new species. In addition to it, our review in the gymnotiform literature brought to light the fact that systematics studies in the order have followed the usual trend in Teleostei, with an inordinate emphasis on osteology and/or external anatomy. As result, other relevant anatomical systems, such as myology, remain mostly unexplored.

Recently, the skeletal musculature has proven to be an important source of phylogenetic information for several bony fish taxa and results indicate that this anatomical system is also fundamental for the reconstruction of the evolutionary tree of this group (e.g., [27, 28, 29]). This scenario inspired us to perform a comparative analysis of the dorsolateral head muscles across Gymnotiformes, which allowed us to recognize new potentially useful characters in phylogenetic studies for Sternopygidae and Eigenmanniinae. Finally, due to the recently contributions on the arrangement of species in *Eigenmannia*, a discussion and a dichotomous key synthesizinng the knowledge on species group within *Eigenmannia* are also provided.

## Materials and methods

Measurements were taken point-to-point to the nearest 0.1 mm with digital calipers as needed under a stereo microscope, preferably on the left side of individuals. Measurements and counts follow [8]. All measurements are presented as proportions of length to end of anal fin, except for subunits of the head, which are given as proportions of head length. Frequencies are given in parentheses after each count and an asterisk indicates counts for the holotype. The holotype was scanned on a 300-kV μ-focus X-ray source micro computed tomography Phoenix v|tome|x m microfocus (General Electric Company) at Laboratório Multiusuário de Processamento de Imagens de Microtomografia Computadorizada de Alta Resolução do Museu de Zoologia da Universidade de São Paulo, Brazil. The scan parameters were set to obtain the maximized spatial resolution and better image contrast. To improve the resolution a Multiscan was made, comprising three individual scans. X-ray projection images were recorded at 1000 ms of time exposure per image, with 90 kV and 185 mA, 1440 images, and voxel resolution of 30 μm. Reconstruction of raw data was performed using the system-supplied software phoenix datos|x reconstruction v. 2.3.0 (General Electric Measurement & Control Solutions, Wunstorf, Germany). Three-dimensional visualization as well as the analysis of the reconstructed data was performed using VGStudio MAX 2.2.3 64 bit (Volume Graphics GmbH, Heidelberg, Germany). The geographic distribution map was prepared using the software Quantum GIS v. 2.14.5.

Specimens were cleared and counterstained according to [30]. Specimens for analysis of musculature were double-stained for cartilages and bones prior dissections following [27]. Vertebral counts included the four vertebrae of the Weberian apparatus. The anatomical terms “transitional vertebrae” and “anterior vertebrae” follows [31,32], respectively. Remaining osteological terminology is based on [8,20,33,34]. Myological nomenclature, including “origin” and “insertion”, follows [35,36], except for sections and subsections of the *adductor mandibulae*, which were grounded in [28,29]. Terminology for cranial nerves follows [37,38] for the “recurrent ramus of anteroventral part of anterior lateral line nerve” (R-Avn) and Freihofer [39] for the “*ramus mandibulars trigeminus*”. The nomenclature of stripes follows [23].

Morphological comparisons of the dorsolateral head muscles were performed between members of all six genera of Sternopygidae (*Archomaelus* Koringa, *Distocyclus* Mago-Leccia, *Eigenmannia*, *Japigny* Meunier, Jégu & Keith, *Rhabdolichops* Eigenmann & Allen and *Sternopygus* Müller & Troschel), as well in members of all other families of Gymnotiformes (Apteronotidae, Gymnotidae, Hypopomidae, Rhamphichthyidae). Putative informative characters are discussed based in the current knowledge on phylogenetic relationships of Sternopygidae (e.g., [15,16,19–21,40–43]).

Most specimens analyzed are deposited in fish collections specified in the Material Examined section. The field studies did not involve endangered species, protected species or areas of conservation. Collection permit was granted by the Instituto Brasileiro do Meio Ambiente e dos Recursos Naturais Renováveis (IBAMA 2621–1) and by the Sistema de Autorização e Informação em Biodiversidade (SISBIO 65628–1). Animal research involving fish at the Museu de Zoologia da Universidade de São Paulo is associated with the project number 226/2015, approved by the Ethics Committee on Animal Use (CEUA) of Instituto de Biologia da Universidade de São Paulo (IB-USP).

Abbreviations used in the text are: CS = cleared and counterstained, msc = specimens dissected for musculature, *L*_EA_ = length to the end of the anal fin, *T*_L_ = total length, *H*_L_ = head length, *C*_FL_ = caudal filament length, and R-Avn = recurrent ramus of anteroventral part of anterior lateral line nerve. Institutional abbreviations follow [44].

## Nomenclatural acts

The electronic edition of this article conforms to the requirements of the amended International Code of Zoological Nomenclature, and hence the new names contained herein are available under that Code from the electronic edition of this article. This published work and the nomenclatural acts it contains have been registered in ZooBank, the online registration system for the ICZN. The ZooBank LSIDs (Life Science Identifiers) can be resolved and the associated information viewed through any standard web browser by appending the LSID to the prefix “http://zoobank.org/”. The LSID for this publication is: urn:lsid:zoobank.org:pub: 98F3AF84-3344-43D2-A751-132F06160C89. The electronic edition of this work was published in a journal with an ISSN, and has been archived and is available from the following digital repositories: PubMed Central, LOCKSS.

## Material examined

In addition to the comparative material examined and listed in [8,9,25,45], the following species were examined: Apteronotidae: *Adontosternarchus balaenops*: MZUSP 83219 (2 msc of 45, 165.2–175.3 mm *L*_EA_), rio Amazonas, Pará, Brazil. *Apteronotus albifrons*: MZUSP 89044 (1 msc of 3, 75.8 mm *L*_EA_), rio Araguaia, Goiás, Brazil. *Orthosternarchus tamandua*: MZUSP 55955 (1 msc of 2, 286.3 mm *L*_EA_), rio Amazonas, Amazonas, Brazil. *Sternarchorhamphus mulleri*: USNM 373030 (1 msc of 5, 222.2 mm *L*_EA_), rio Negro, Amazonas, Brazil. Gymnotidae: *Gymnotus* gr. *carapo*: MPEG 3012 (1 msc of 4, 232.2 mm *L*_EA_), rio Turiaçu, Maranhão, Brazil; *Gymnotus* gr. *pantherinus*: MZUSP 113616 (1 msc of 4, 151.3 mm *L*_EA_), rio Cubatão, São Paulo, Brazil. *Gymnotus cylindricus*: USNM 134701 (1 msc of 23, 178.5 mm *L*_EA_), Río Hondo, Guatemala. Hypopomidae: *Brachyhypopomus beebei*: MZUSP 103275 (1 msc of 9, 74.6 mm *L*_EA_), rio Jari, Amapá, Brazil. *Hypopomus artedi*: USNM 408442 (1 msc of 2), 202.7 mm *L*_EA_, Paloemeu River, Suriname. Rhamphichthyidae: *Gymnorhamphichthys rosemariae*: MZUSP 56317 (1 msc of 2, 116.3 mm *L*_EA_), rio Negro, Amazonas, Brazil. *Hypopygus lepturus*: MPEG 10169 (1 msc of 9, 61.0 mm *L*_EA_), rio Amazonas, Pará, Brazil. *Rhamphichthys depranium*: MZUSP 36144 (1 msc of 2), 282.3 mm *T*_L_ (damaged), Lago Amanã, rio Japurá, Amazonas, Brazil. *Rhamphichthys hahni*: MZUSP 24736 (1 msc, 479.5 mm *T*_L_) (damaged), rio Caxipó da Ponte, Mato Grosso, Brazil; *Rhamphichthys marmoratus*: MPEG 8833 (1 msc, 65.8 mm *H*_L_; damaged), rio Amazonas, Pará, Brazil. Sternopygidae: *Archolaemus orientalis*: MPEG 21509 (1 msc, paratype, 110 mm *L*_EA_), rio São Francisco, Minas Gerais, Brazil. *Distocyclus conirostris*: MZUSP 23316 (1 msc of 3, 242.2 mm *L*_EA_), rio Solimões, Amazonas, Brazil. *Eigenmannia oradens*: ANSP 190768 (1 msc of 6, paratype, 101.4 mm *L*_EA_), rio Ventuari, Amazonas, Venezuela. *Eigenmannia antonioi*: MPEG 29487 (1 msc of 11, 80.0 mm *L*_EA_), rio Anapu, rio Amazonas, Pará, Brazil. *Eigenmannia besouro*: MZUSP 98748, 1 paratype of 2, 89.2 mm *L*_EA_), rio São Francisco, Bahia, Brazil. *Eigenmannia correntes*: MNRJ 46334 (6 of 31 paratypes, 4, 76.6–103.9 mm *L*_EA_ + 2 CS, 66.6–94.0 mm *L*_EA_); MNRJ 46335 (5 of 21 paratypes, 59.6–75.4 mm *L*_EA_), Córrego de Baixo, left margin tributary of rio Correntes. *Eigenmannia humboldtii*: FIELD 56812 (1 msc of 7, 186.2 mm *L*_EA_), Puerto del Rico, Colombia. *Eigenmannia limbata*: MZUSP 75569 (1 msc of 2, 160.0 mm *L*_EA_), Lago da Terra Preta, rio Negro, Amazonas, Brazil. *Eigenmannia loretana*: MZUSP 26014 (3, 68.5–99.4 mm *L*_EA_), Loboccocha, Masisea, Dpto. Ucayali, Perú. *Eigenmannia macrops*: USNM 405266 (1 msc of 16, 103.2 mm *L*_EA_), Cuyuni River, Guyana. *Eigenmannia meeki*: MZUSP 119018 (1 msc of 2, paratype), 160.2 mm *L*_EA_, Río Pucuro, Panamá. *Eigenmannia trilineata*: MZUSP 111146 (1 msc, 305.0 mm *L*_EA_), rio de La Plata, Argentina. *Japigny kirschbaum*: FIELD 50185 (1 msc of 16, 137.2 mm *L*_EA_), Itabu Creek, New River Drainage, Guyana. *Rhabdolichops lundbergi*: INPA 11406 (1 msc of 10, 110.2 mm *L*_EA_), rio Coari, Amazonas, Brazil. *Rhabdolichops troscheli*: MZUSP 57704 (2 msc of 79, 122.2–140.2 mm *L*_EA_), rio Negro, Amazonas, Brazil. *Rhabdolichops zareti*: CAS 57444 (1 msc of 37, 88.9 mm *L*_EA_), Río Orinoco, La Providencia, Venezuela. *Sternopygus astrabes*: MZUSP 88795 (1 msc of 2, 151.0 mm *L*_EA_), rio Preto da Eva, Amazonas, Brazil. *Sternopygus macrurus*: MZUSP 32215 (1 of 13, 212.6 mm *L*_EA_), rio Amapá, Amapá, Brazil. MPEG 22756 (2 msc of 5, 240.4–245.8 mm *L*_EA_), rio Japurá, Amazonas, Brazil. *Sternopygus xingu*: MPEG 8657 (1 msc, 230.5 mm *L*_EA_), rio Amazonas, Pará, Brazil.

## Results

*Eigenmannia sirius*, sp. nov. urn:lsid:zoobank.org:act: FC545922-CDC8-45C0-9287-593B9ABEEC18

(Figs 1–8)

**Fig 1:**
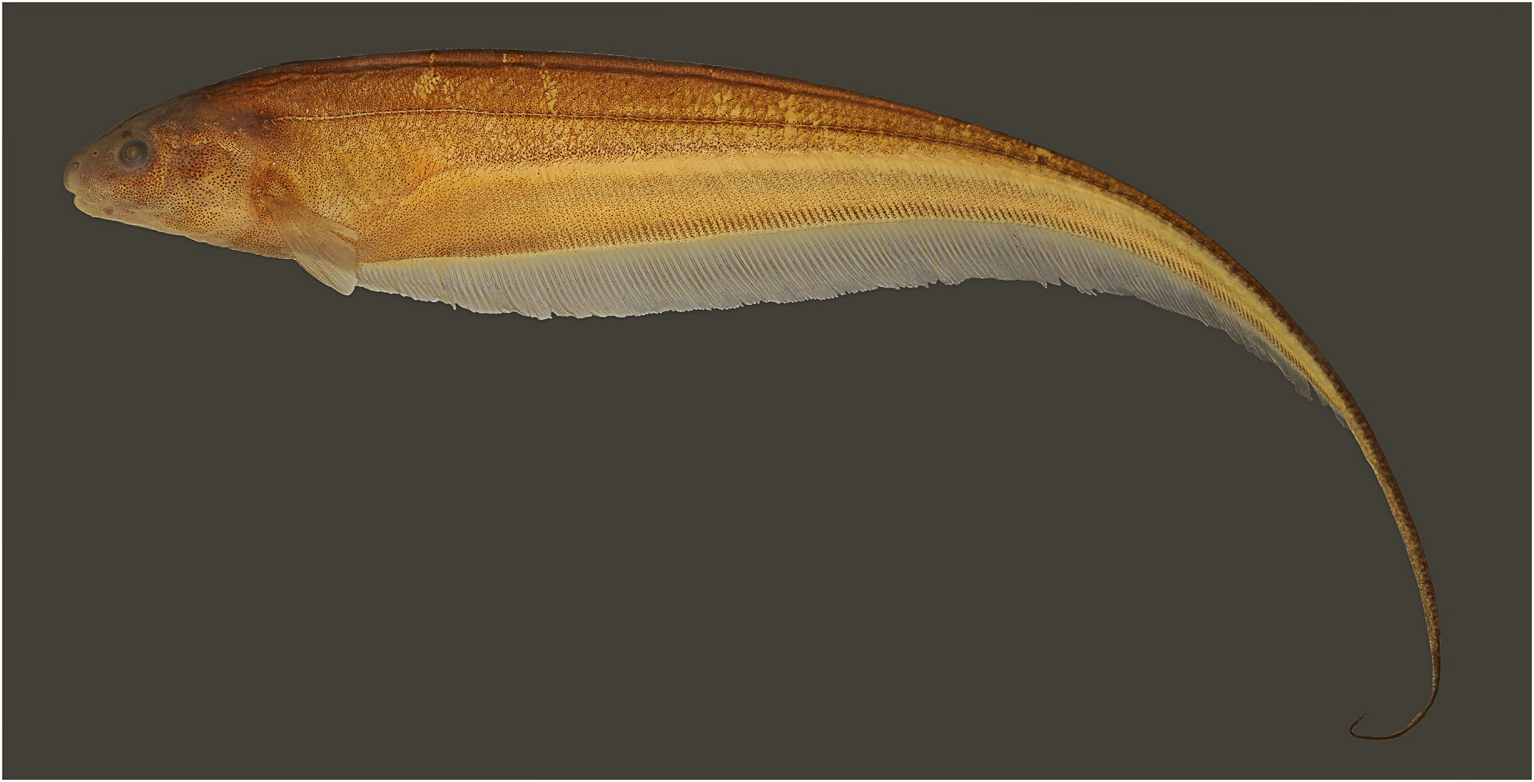
Lateral view of *Eigenmannia sirius*, MZSUP 121668, holotype, 127.5 mm *L*_EA_, Mato Grosso, Brazil, rio Mutum, tributary of rio Juruena, rio Tapajós basin.

**Fig 2:**
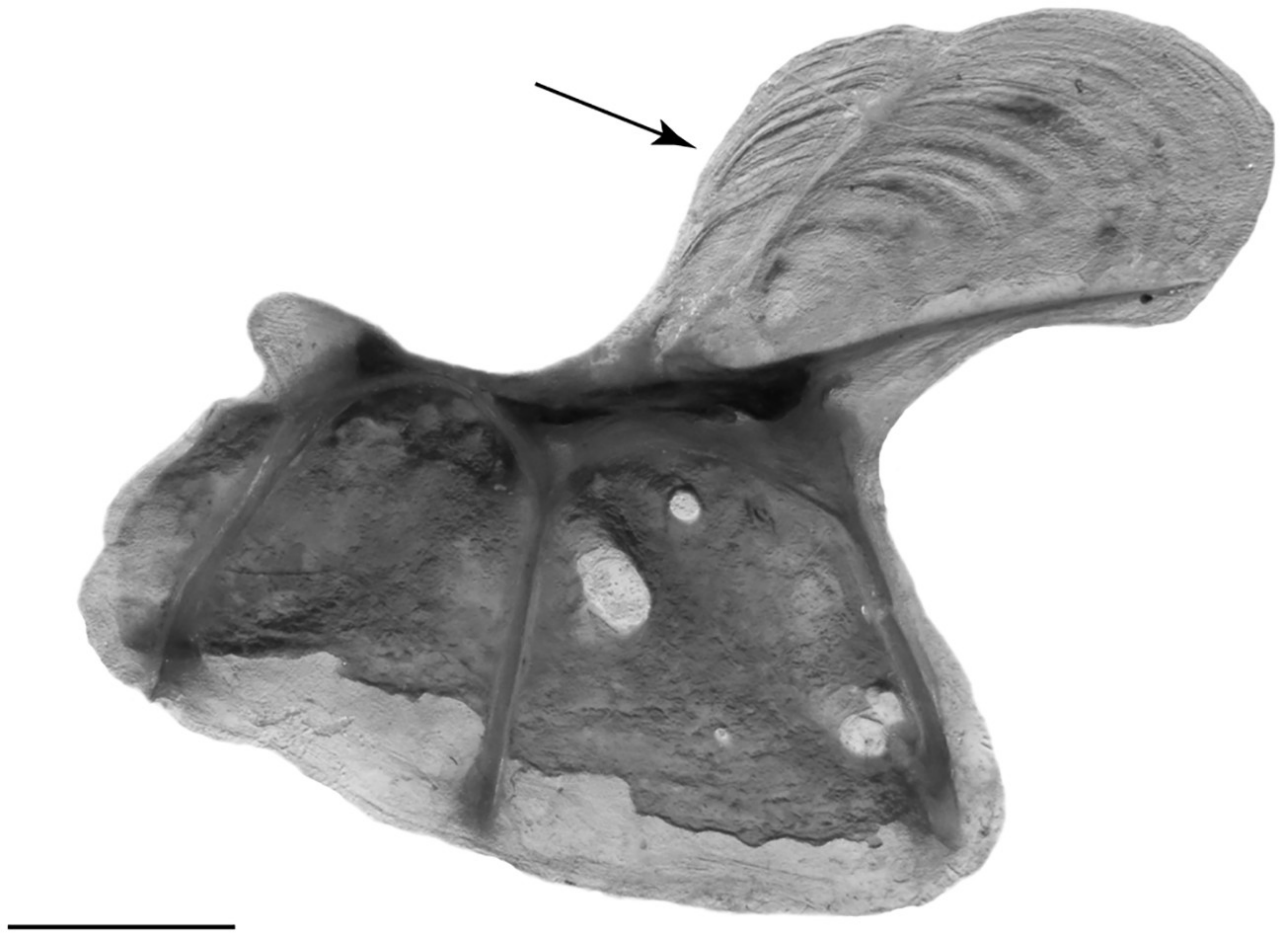
Infraorbital 1+2 of *Eigenmannia sirius*, MZUSP 118582, 93.3 mm *L*_EA_, paratype. Arrow indicates the posterodorsal expansion. Scale bar = 1 mm.

**Fig 3:**
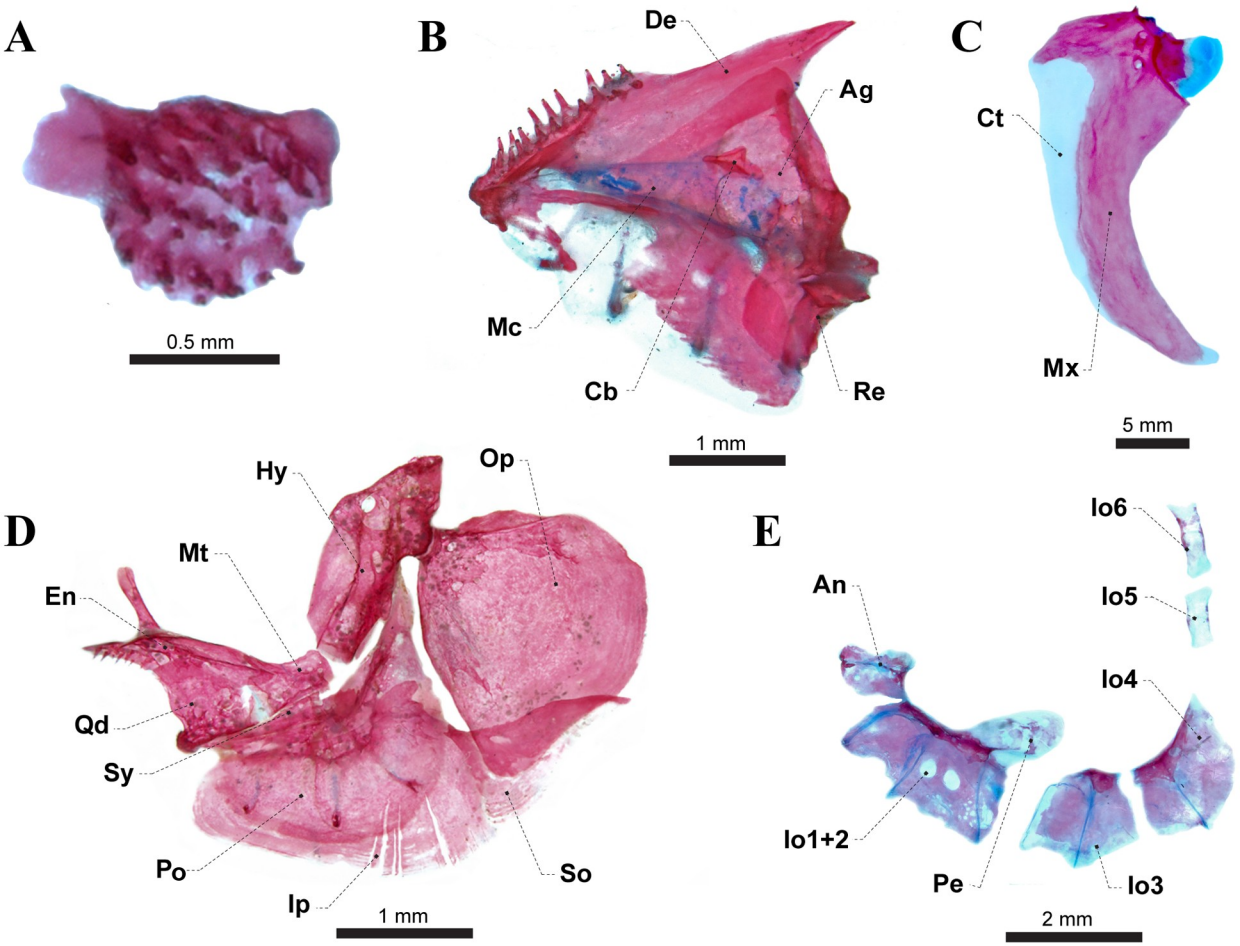
Osteological elements of *Eigenmannia sirius*: A) premaxilla (MCP 41099, 88.7 mm *L*_EA_); B) dentary, C) maxillary bone (MZUSP 118582, 93.3 mm *L*_EA_); D) suspensorial bones (MZUSP 118581, 110.5 mm *L*_EA_); E) infraorbital series (MCP 41099, 88.5 mm *L*_EA_). Ag = anguloarticular; An = antorbital; Cb = coronomeckelian bone; Ct = connective tissue; De = dentary; En = endopterygoid; Hy = hyomandibula; Io = infraorbital; Ip = interopercle; Mt = Metapterygoid; Mx = maxillary bone; Mc = meckel’s cartilage; Op = opercle; Pe = posterodorsal expansion of Infraorbitals 1+2; Po = preopercle; Qd = quadrate; Re = retroarticular; So = subopercle; Sy = symplectic bone.

**Fig 4:**
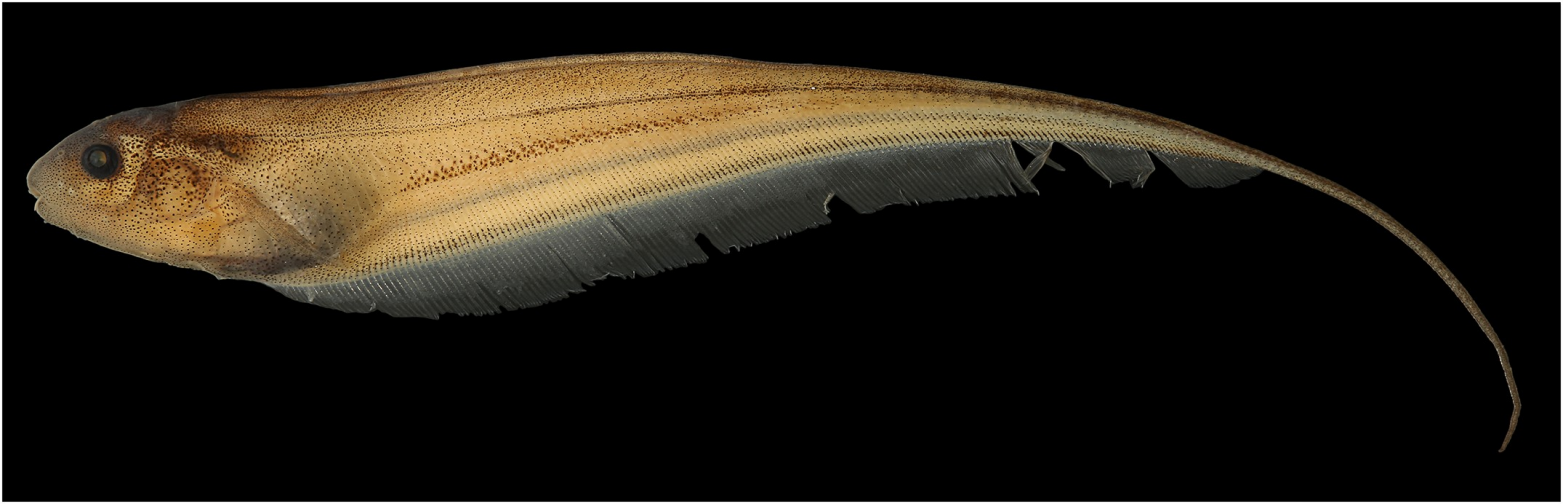
Lateral view of *Eigenmannia sirius*, MCP 41099, paratype, 75.6 mm *L*_EA_, Mato Grosso, Brazil, rio Mutum, tributary of rio Juruena, rio Tapajós basin.

**Fig 5:**
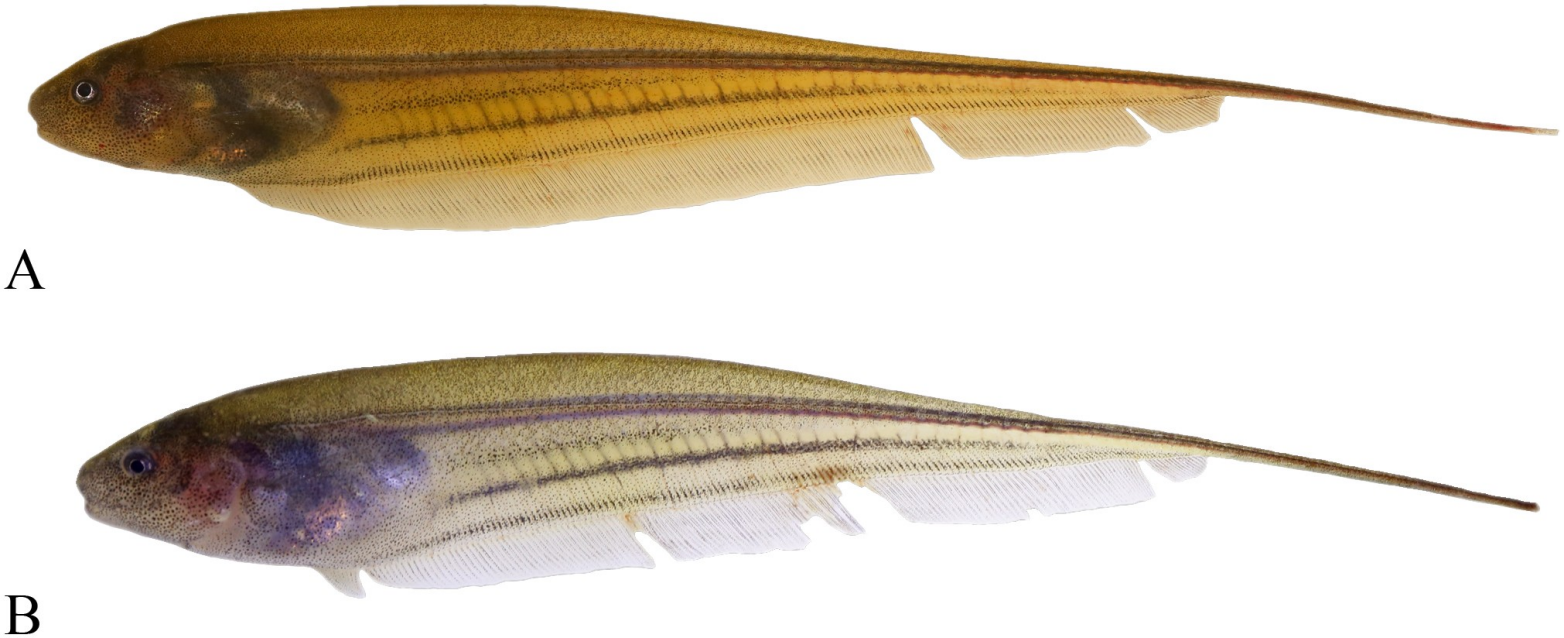
Color in life of *Eigenmannia sirius*, MZUSP 123938, paratypes, 93.7 and 77.3 mm *L*_EA_, A and B respectively, rio Mutum, rio Juruena, Mato Grosso, Brazil. Caudal filament damaged.

**Fig 6:**
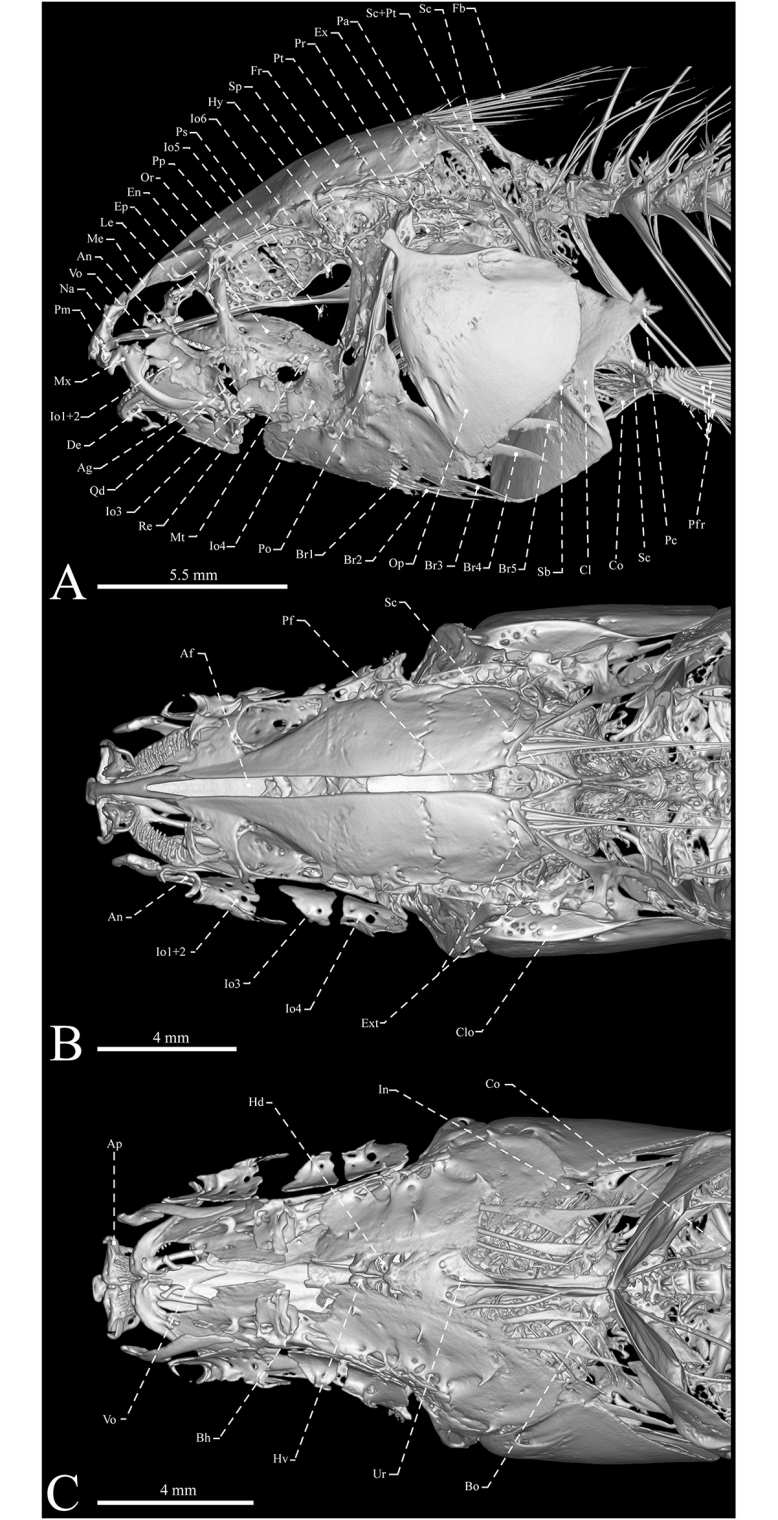
Computed tomography renderings of anterior portion in lateral (A), dorsal (B) and ventral views (C) of *Eigenmannia sirius*, MZSUP 121668, holotype, 127.5 mm *L*_EA_. Af = anterior fontanelle; Ag = anguloarticular; Ao = antorbital; Ap = anterolateral process of premaxilla; Bh = basihyal; Bo = basioccipital; Br = branchiostegal rays; Cl = Cleithrum; Clo = crest of the *levator arcus palatini*; Co = coracoid; De = dentary; En = endopterygoid; Ep = endopterygoid process; Ex = exoccipital; Ext = extrascapular; Fb = filamentous bone; Fr = frontal; Hd = hypohyal dorsal; Hv = hypohyahl ventral; Hy = hyomandibula; In = Interopercle; Io = infraorbitals; Le = lateral ethmoid; Me = mesethmoid; Mt = metapterygoid; Mx = maxillary bone; Na = nasal; Op = opercle; Or = orbtosphenoid; Pa = parietal; Pc = postcleithrum; Pfr = pectoral-fin rays; Pf = posterior fontanelle; Pm = premaxilla; Po = preopercle; Pp = parasphenoid; Pr = prootic; Ps = pterosphenoid; Pt = pterotic; Qd = quadrate; Re = retroarticular; So = subopercle; Sca = scapula; Sc+Pt = suptracleithrum+posttemporal; So = supraoccipital; Sp = sphenotic; Ur = urohyal; Vo = vomer.

**Fig 7:**
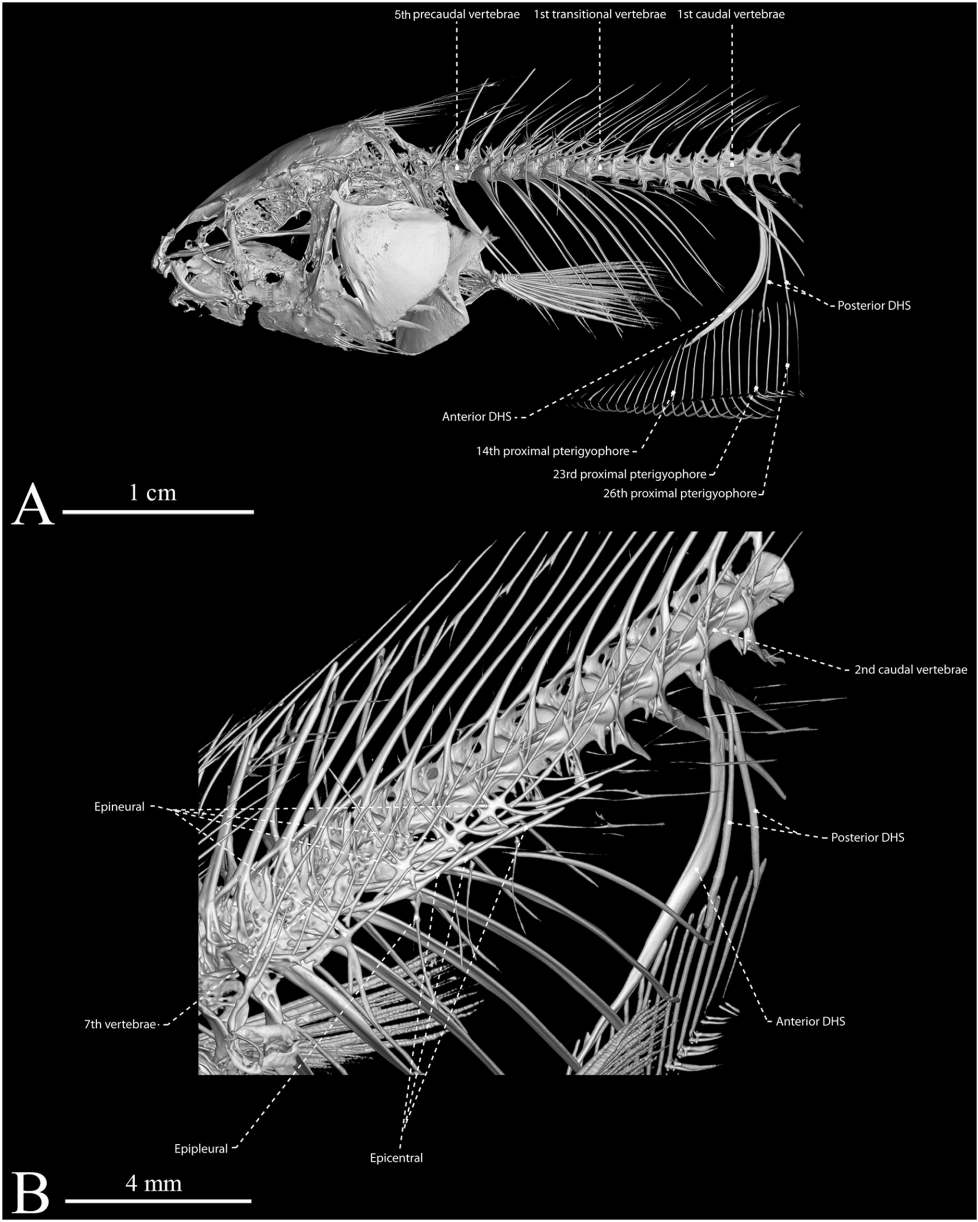
Computed tomography renderings of anterior portion in left lateral (A) and right inverted oblique (B) views of *Eigenmannia sirius*, MZSUP 121668, holotype, 127.5 mm *L*_EA_.

**Fig 8:**
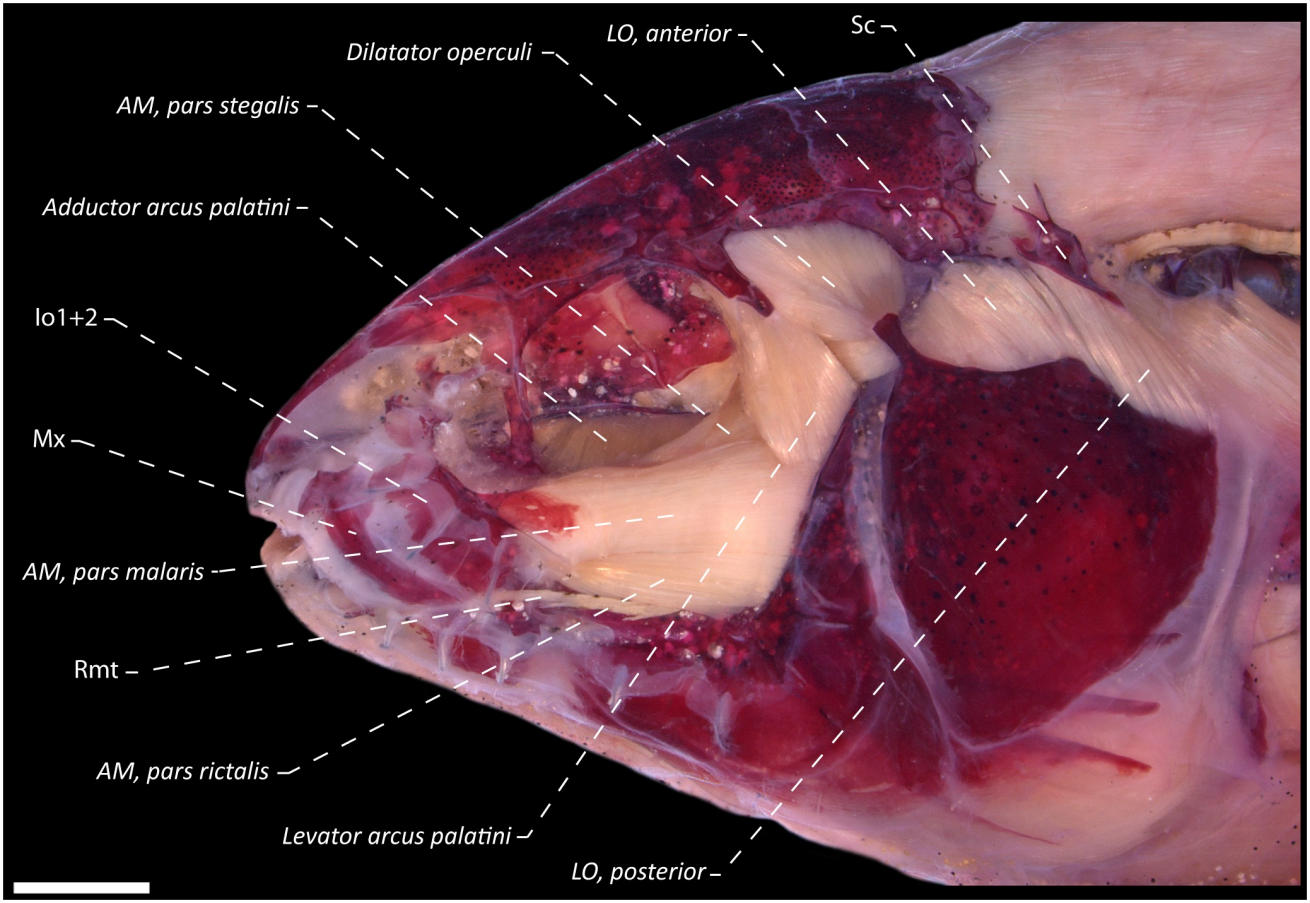
Lateral view of the dorsolateral head muscles of the *Eigenmannia sirius*, MCP 41099, paratype, 97.6 mm *L*_EA_. AM = *adductor mandibulae*; Io = infraorbitals; LO = *levator operculi*; Mx = maxillary bone; Rmt = *ramus mandibularis trigeminus*; Sc = supracleithrum canal.

*Eigenmannia* sp. n. Ohara & Loeb [46] (checklist).

Holotype: MZUSP 121668 (127.5 mm *L*_EA_), Comodoro, Mato Grosso, Brazil, rio Mutum, tributary of upper rio Juruena, rio Tapajós basin, 13°05’08”S 59°53’32”W, W.M. Ohara, B. Barros & D.H. Hungria, 25 Jul 2013.

Paratypes: All from rio Mutum, upper rio Tapajós, Comodoro, Mato Grosso, Brazil: FMNH 141416 (1, 123.7 mm *L*_EA_). MCP 41099 (26, 51.6–126.8 mm *L*_EA_ + 1 msc, 97.6 mm *L*_EA+_ 3 CS, 54.7–88.7 mm *L*_EA_), P. Lehmann, V. Bertaco, J. Pezzi & F. Lima, 22 July 2004. MZUSP 123938 (7, 51.0–93.7 mm *L*_EA_), W.M. Ohara, 3 Aug 2018. MPEG 34989 (1, 92.3 mm *L*_EA_). MPEG 34990 (1, 88.3 mm *L*_EA_). MZUSP 118579 (2, 102.7–106.2 mm *L*_EA_). MZUSP 118580 (2, 87.6–115.9 mm *L*_EA_). MZUSP 118581 (6, 60.7–85.5 mm *L*_EA_ + 2 CS, 58.8–110.5 mm *L*_EA_). MZUSP 118582 (10, 86.6–120 mm *L*_EA_ + 2 CS, 83.8–93.3 mm *L*_EA_).

### Diagnosis

*Eigenmannia sirius* is diagnosed from all putative congeners with exception of *Eigenmannia trilineata* species-group (*sensu* [8]), by the presence of superior midlateral stripe (*vs*. absence). The new species differs from all species of the *Eigenmannia trilineata* species-group, except *E*. *besouro*, *E*. *correntes*, *E*. *vicentespelaea*, and *E*. *waiwai*, by the subterminal mouth (*vs*. terminal). It differs from *E*. *besouro*, *E*. *correntes*, *E*. *vicentespelaea*, and *E*. *waiwai* by relative depth of posterodorsal expansion on infraorbitals 1+2 corresponding to 70% length of infraorbitals 1+2 (Fig 2—*vs*. 40% in *E*. *besouro*, *E*. *correntes* and *E*. *waiwai*, and approximately equals total length of infraorbitals 1+2 in *E*. *vicentespelaea*), and in having 15 precaudal vertebrae (*vs*. 14 in *E*. *besouro* and *E*. *correntes*, 13–14 in *E*. *vicentespelaea* and 12–13 in *E*. *waiwai*). *Eigenmannia sirius* can be differentiated from *E*. *besouro* by the origin of the superior midlateral stripe at vertical between base of 23^rd^ to 31^st^ anal-fin ray (*vs*. origin at vertical between 5^th^ to 15^th^ anal-fin ray). The new species is also diagnosed from *E*. *correntes* by the eye diameter (17.2–23.8% *H*_L_ *vs*. 10.6–13.3% *H*_L_), and by the mouth width (13.1–22.4% *H*_L_ *vs*. 23.5–26.0% *H*_L_). *Eigenmannia sirius* is further distinguished from *E*. *vicentespelaea* and *E*. *waiwai* by having 15–24 premaxillary teeth (Fig 3A *vs*. 25–26 and 35–40, respectively) and 15–33 dentary teeth (Fig 3B *vs*. 38–45 and 37–38, respectively). It additionally differs from *E*. *vicentespealea* by having 9–12 scale rows above lateral line (*vs*. seven or eight) and the coronomeckelian bone corresponding to 20–25% of length of Meckel’s cartilage (Fig 3B *vs*. 45%). The new species can be distinguished from *Archolaemus* species by the eye completely covered by thin membrane (*vs*. a free orbital rim); from *Distocyclus* by the rounded snout in profile (*vs*. conical snout); from *Japigny* by the absence of alternating dark bands on flanks (*vs*. presence); and from *Rhabdolichops* by the region above the lateral line on the anterior portion of the body covered by scales (*vs*. absence of scales above the lateral line on the anterior portion of the body).

### Description

Body shape and pigmentation in Figs 1, 4 and 5. Morphometric data for examined specimens in Table 1. Largest examined specimen 127.5 mm *L*_EA_. Body elongate, distinctly compressed. Greatest body depth at vertical through distal margin of pectoral fin. Dorsal profile of body convex to straight. Ventral profile slightly convex. Caudal filament elongated, about a half of the length to end of anal-fin in adult specimens.

**Table 1:** Morphometrics for examined specimens of *Eigenmannia sirius*, new species. SD = Standart Deviaton.

	Holotype	Min	Max	N	Mean	SD
*T*_L_ (mm)	165.7	83.5	165.7	24	-	-
*L*_EA_ (mm)	127.5	60.7	127.5	27	-	-
*H*_L_ (mm)	16.5	9.1	17.0	27	-	-
*C*_FL_ (mm)	38.2	18.3	47.9	24	-	-
**Percent of *L***_**EA**_						
Total length	130.0	117.3	149.7	24	135.5	7.7
Caudal filament length	30.0	17.3	49.7	24	35.5	7.7
Greatest body depth	14.3	14.0	18.8	26	15.9	1.2
Body depth at anal-fin origin	14.2	12.7	16.9	26	14.9	1.0
Body width	6.1	5.7	8.8	26	7.1	0.7
Preanal-fin distance	18.1	17.3	21.4	26	19.1	1.0
Prepectoral-fin distance	14.9	14.1	18.1	26	15.9	0.9
Anal-fin length	82.1	74.5	85.1	26	81.1	2.3
Pectoral-fin length	9.7	8.2	11.3	26	10.1	0.8
Snout to anus	9.2	7.1	14.8	26	10.9	2.1
Head length	12.9	12.9	17.2	26	14.8	0.9
**Percent of *H***_**L**_						
Head width at opercle	58.8	49.7	59.0	27	54.0	2.6
Head width at eye	47.1	37.7	51.6	27	43.5	3.4
Head depth at nape	79.3	70.2	83.4	27	77.0	3.4
Head depth at eye	53.7	52.0	63.5	27	56.2	2.7
Snout length	32.2	25.4	35.7	27	32.3	2.1
Snout to posterior nostril	22.1	19.0	25.0	27	22.6	1.4
Posterior nostril to eye	10.0	6.4	11.3	27	8.9	1.4
Postorbital distance	56.9	48.6	56.9	27	52.7	2.2
Branchial opening	29.1	26.2	33.9	27	30.1	2.0
Internarial width	18.1	14.6	20.3	27	17.8	1.5
Internarial distance	9.8	8.3	17.4	27	11.1	2.7
Interorbital distance	27.9	26.2	37.2	27	31.3	2.9
Eye diameter	19.6	17.2	23.8	27	20.5	1.5
Mouth length	19.8	16.4	23.1	27	19.6	1.9
Mouth width	16.4	13.1	22.4	26	17.3	1.8
**Percent of *C***_**FL**_						
Caudal filament width	1.9	1.1	4.2	22	2.0	0.7
Caudal filament depth	5.1	2.8	7.5	22	3.9	1.1

Head compressed, greatest width at opercular region and greatest depth at nape. Dorsal profile of head convex. Ventral profile of head slightly straight. Snout subconical in lateral view. Mouth subterminal with rictus extending posteriorly to vertical between nares. Anterior naris tube-like, closer to snout tip than to anterior margin of eye. Posterior naris rounded, without tube; near midpoint between anterior naris and anterior margin of eye. Eye small, circular, completely covered by thin membrane, on anterior one-half of *H*_L_ and dorsolaterally positioned. Gill opening limited to posterior margin of opercle and extending above and below pectoral-fin base. Gill rakers tiny and fleshy. Branchial membranes joined at isthmus.

Anus and urogenital papilla adjacent. Position of anus and urogenital papilla shifting through ontogeny from vertical through posterior margin of opercle to vertical through posterior margin of eye.

Scales small, cycloid, extending from immediately posterior of head to tip of caudal filament. Scales present on mid-dorsal region of body. Scales above lateral line at midbody nine (1), 10* (11), 11 (13) or 12 (1). Lateral-line scales to vertical through anal-fin terminus 92 (1), 94 (1), 96 (1), 97 (1), 100 (1), 103 (3), 104 (4), 105 (2), 106* (3), 107 (3), 110 (1), 111 (2) or 114 (1).

Pectoral-fin rays ii,13 (7), ii,14* (12) or ii,15 (8). Distal pectoral-fin margin straight and surpassing anal-fin origin. Total anal-fin rays 157 (1), 158 (1), 159 (1), 160 (1), 165 (2), 166 (1), 168 (2), 169 (2), 170 (1), 171 (1), 172 (1), 174* (2), 176 (5) or 183 (1). Anal-fin origin at vertical through posterior limit of pectoral-fin base. Distal margin of anal fin straight. First unbranched rays tiny; rays progressively increasing in size to first branched rays. Branched rays nearly equal length, except for posterior most rays that progressively decrease in size.

## Relevant osteological features of *Eigenmannia sirius*

Mesethmoid oriented at about 45° angle from vomer, until reaching anterior margin of frontals; anterior portion with small lateral process on each side (Fig 6A). Paired frontals convex in lateral profile, about 75% of length of skull. Anterior portion of anterior fontanelle limited by contralateral posterior processes of mesethmoid and completely surrounded by frontals. Posterior fontanelle about 80–90% length of anterior fontanelle. Anterior one third of posterior fontanelle surrounded by frontals, posterolateral portion by parietals and posterior edge by supraoccipital. Parietals form lateral margins of posterior fontanelle and contact frontals anteriorly, supraoccipital posteriorly, epioccipitals and pterotics laterally (Fig 6B).

Vomer arrow shaped anteriorly, with small anterior processes on each side; becoming larger posteriorly and diverging in two posterolateral process, contacting anterior margin of parasphenoid. Parasphenoid larger than posterior portion of vomer, with a long crest that serves as a site of origin of *adductor arcus palatini*. Anteriorly, parasphenoid reaches half portion of vomer, posteriorly surrounding anteroventral margin of prootics and ventral surface of basioccipital. At its posterolateral portion, parasphenoid contacts posteroventral margin of pterosphenoid through a tapered lateral process; and dorsally contacts orbitosphenoid entirely.

Supraoccipital contacts parietals anteriorly and epioccipitals posterolaterally. Epioccipitals form posterodorsal corners of neurocranium and contacts supraoccipital medially. Exoccipital contacts basioccipital ventrally, pterotic anterodorsally, prootics anteriorly, and epioccipitals posterodorsally. Internally, basioccipital and exoccipitals form a pair of chambers for *cf*. *asteriscus* otoliths; and pterotics and prootics allocates a pair of *cf*. *lapillus* otoliths located anterodorsally to basioccipital and exoccipitals chambers. Pterotics form posterolateral portions of skull roof, and contacts prootics and exoccipitals ventrally, epioccipitals posteriorly, parietals dorsally, frontals anterodorsally and sphenotics anteriorly. Prootics contacts basioccipital, exoccipitals, pterotics, sphenotics and pterosphenoids through cartilage filled sutures, and directly contacts to parasphenoid. Prootic and exoccipital with prominent foramenae. Supraoccipital extending dorsally to dorsal margin of parietals.

Orbitosphenoid connected dorsally to neurocranium and posteriorly separated from pterosphenoid by a segment of cartilage. Entire ventral surface of orbitosphenoid contacting dorsal margin of parasphenoid. Pterosphenoid contacts orbitosphenoid anteriorly, and associates with frontal dorsally. Pterosphenoid contacts parasphenoid only posteroventrally, with its anteroventral surface not contacting dorsal margin of parasphenoid, forming a lateral fenestra. Lateral ethmoids as small elements Y-shaped that are positioned in a vertical thought contact between mesethmoid and frontals; connected ventrally to parasphenoid by a connective tissue and to frontals by two strong and short ligaments.

Premaxilla somewhat rectangular with anterolateral process about one third of anterior margin of bone. Premaxillary teeth 15 (1), 20 (1), 21 (2), 22 (1), 23* (1) or 24 (1) arranged in three (3) or four* (2) rows (Fig 3A). Dentary triangular with 15 (1), 18 (1), 19 (1), 21 (1), 22 (2), 23 (1), 25 (1) or 33* (1) teeth arranged in two (8) or three* (1) rows; all similar in size (Fig 3B). Dentary with three bony arches, which comprises a robust laterosensory canals ossified along lateroventral surface. Coronomeckelian bone corresponds to 20–25% of length of Meckel’s cartilage (Fig 3B). Anguloarticular with one bony arch and a narrow crest on posterolateral surface for *adductor mandibulae*, *pars rictalis* insertion, extending dorsally; small rectangular process on medial surface connecting to Meckel’s cartilage. Retroarticular small, roughly rectangular, located at posteroventral margin of anguloarticular. Maxilla edentulous, slender and slightly curved posteriorly, with short hook-shaped anterodorsal process equal to width of posterior nostril in adult specimens (Fig 3C); and equivalent to two-thirds of posterior nostril in juveniles, increasing in size thought ontogeny (abnormal formation in one specimen, without process in one side—MCP 41099). Posterior margin of maxilla reaching posterior margins of Io1+2. Cartilaginous autopalatini connecting endopterygoid to posterior cartilage of maxilla. Endopterygoid roughly triangular with well-developed dorsally directed process, equal to half of length this bone, attached to anterodorsal portion of orbitosphenoid. Endopterygoid with small, pointed, conical teeth arranged in one (4) or two (3) rows at anterior portion. Endopterygoid teeth nine (3), 10 (1), 11 (1), 12 (1) or 13 (1) (Fig 3D).

Base of quadrate roughly trapezoidal in shape extending into pointed triangular shape anterodorsally, articulating with preopercle and sympletic at base through posteroventral process; its condyle extending anteroventrally from base and articulating with retroarticular and anguloarticular. Metapterygoid slightly rectangular, without posterodorsal process. Preopercle crescent-shaped, with five bony arches that corresponds to laterosensory canal tubes along lateral surface. Interopercle teardrop-shaped, with posterodorsal expansion and margins rounded. Opercle roughly triangular, dorsal margin convex, with a pointed anterodorsal process that anchors *dilatator operculi* and a medially expansion forming a crest for *levator operculi* insertion. Subopercle sickle-shaped, tapering posterodorsally, forming concave dorsal profile. Subopercle and interopercle becoming membranous distally. Hyomandibula at roughly 90° to horizontal line through long axis of head; dorsal articulating head roughly one and a half time wider than ventral margin; laminar anterior shelf from widest hyomandibula point to anteroventral margin, which serves as site of *adductor arcus palatini* insertion (Figs 3D and 6). Neurocranial articulatory head of hyomandibula pointed; opercular articulatory head distinct from main body of hyomandibula and directed posteroventrally. Foramen for hyomandibular trunk of facial nerve positioned anterodorsally, near base of neurocranial articulatory head. Second foramen for a branch of this nerve reduced and located near dorsal portion of preopercle; third foramen, located anteroventrally in relation of second foramen that serves for exit of *ramus mandibularis trigeminus*. All foraminae positioned entirely within body of hyomandibula. Sympletic elongate and triangular, located in a medial crest of preopercle and posteroventral portion of quadrate (Fig 3D).

Supraorbital canal robust, forming a highly ossified shelf-like structure. Nasals present. Antorbital and infraorbitals 1+2 to 4 enlarged, partially cylindrical. Fifth and sixth infraorbitals slender and tubular. All infraorbitals and antorbital with slender osseous arches, except for Io1+2, that presents three bony arches. Antorbital 85–90% of total length of Io1+2. Depth of posterodorsal expansion on infraorbitals 1+2 near 70% length of infraorbitals 1+2. Infraorbitals 3 and 4 closely associated (Figs 2, 3E and 6).

Six (1) or seven (1) gill rakers on first ceratobranchial. Four (2) gill rakers on first infrapharyngobranchial. Lower pharyngeal plate with eight (1) or 14 (2) teeth; upper pharyngeal plate with six (1), seven* (2) or eight (1) teeth. Branchiostegal rays five (5). First and second branchiostegal rays narrow. Third to fifth branchiostegal rays spatulate. First to fourth branchiostegal rays attached to anterior ceratohyal. Fifth branchiostegal ray attached to posterior ceratohyal (Fig 6).

Posttemporal bones fused with supracleithrum, and lie at posterolateral surface of skull at epioccipitals and pterotic sutures. Scapula broad, visible laterally, and contacts mesocoracoid anterodorsally and anteroventrally, and coracoid ventrally. Scapular foramen present. Supports for pectoral fin include propterygium and three proximal radials (proximals three and four co-ossified).

Anal pterygiophores slender and thin. Each fin ray contacts its proximal radial, its distal radial, and posterior distal radial. Precaudal vertebrae 15 (8). Transitional vertebrae four (2) or five* (6). Vertebrae to end of anal fin 65 (1) or 66 (2). Pleural ribs seven (3) or eight* (4). Displaced hemal spines three (8) (Fig 7).

## Relevant features of dorsolateral head muscles of *Eigenmannia sirius*

Dorsolateral head muscles illustrated in Fig 8. Three primary sections of *adductor mandibulae*, *segmentum facialis* well differentiated, except mesialmost fibers of *pars malaris* and *pars rictalis* which are partially continuous with lateroventral fibers of *pars stegalis*. *Adductor mandibulae*, *pars malaris* originates from preopercle and hyomandibula, and inserts primarily by a fibrous attachment to medial face of posterodorsal expansion on infraorbitals 1+2. At portion near insertion, mesialmost fibers converges into a poorly differentiated endomaxilar ligament which, in turn, inserts on posteromedial portion of maxilla. *Adductor mandibulae*, *pars malaris* located laterally to dorsal portion of *pars rictalis* and lateroventrally to *pars stegalis*. *Adductor mandibulae*, *pars rictalis* arises from quadrate, symplectic, preopercle and hyomandibula, with its posterolateral fibers restricted to anterior margin of preopercle fossa. Insertion of *pars rictalis* occurs mostly on the coronoid process, however, with some lateral fibers attached to buccopalatal membrane and posterior margin of anguloarticular. *Adductor mandibulae*, *pars stegalis* originates from anterior margin of hyomandibula, metapterygoid and quadrate, and converges anteriorly onto a meckelian tendon that attaches to coronomeckelian tendon, with some anterodorsal fibers associated to mandibular tendon. *Adductor mandibulae*, *pars stegalis* located laterally only to midposterior and posterior portion of *adductor arcus palatini*, resulting in a partial overlap. All sections of *segmentum facialis* are fibrous, without intermuscular bones.

*Adductor mandibulae*, *segmentum mandibularis* present as a single component, arising from mandibular tendon and attached to medial face of dentary and anguloarticular. *Adductor mandibulae*, *segmentum mandibularis* restricted to dorsal portion of the Meckel’s cartilage, extending about 60% of dorsal margin of this cartilage. Nerve *ramus mandibularis trigeminus* lies laterally to *pars stegalis* and medially to *pars malaris* and *rictalis*, with an unidentified branch medial to a set of fibers of midposterior portion of *pars rictalis*.

*Levator arcus palatini* approximately triangular in shape, arising from sphenotic bone and inserting on anterodorsal margin of hyomandibula. *Levator arcus palatini* origin narrower than its extension at site of insertion, about half of its insertion. *Levator arcus palatini* in a single mass of fibers, with four discernible sets of fibers near insertion, located distinctly to *pars malaris*: anterolateral and anteromedial set of fibers medial to *pars malaris*; posterolateral and posteromedial set of fibers lie lateral to *pars malaris*. At dorsal portion, posterolateral *levator arcus palatini* fibers located parallel to anterior portion of *dilatator operculi*. *Dilatator operculi* nearly conical in shape, located posteriorly to *levator arcus palatini*. This muscle arises from sphenotic, pterotic and hyomandibula, and its insertion occurs invariably on dorsal process of opercle.

*Levator operculi* muscle, superficial and located posteriorly to *dilatator operculi* and differentiated in two sections. *Levator operculi anterior* arises from pterotic and hyomandibula; *levator operculi posterior* originates from supracleithrum canal with both sections inserting on opercle. R-Avn lies medial to entire *levator operculi*. *Adductor arcus palatini* originates from parasphenoid and prootic. At anterior portion, *adductor arcus palatini* inserts on lateral face of endopterygoid and metapterygoid; towards posterior portion, this muscle inserts on mesial face of hyomandibula.

The *adductor hyomandibulae* muscle relatively well differentiated from posterior portion of *adductor arcus palatini*, arising from prootic and pterotic, inserting on posteromedial face of hyomandibula. *Adductor operculi* originates from pterotic, prootic and exoccipital, and its insertion occurs on medial face of opercle (Fig 8).

### Color in alcohol

Ground coloration of body beige (Figs 1 and 4). Body densely covered by dark brown chromatophores gradually more spaced ventrally. Chromatophores more concentrated on perforated scales forming a narrow lateral line stripe. Superior midlateral stripe thick, one or one and half scales deep, tapering from vertical between base of 23^rd^ to 31^st^ anal-fin ray to posterior two-thirds of body. Second layer of pigmentation formed by multiple, small bars of dark chromatophores situated between musculature associated with anal-fin pterygiophores. Dark individual bars in combination form two stripe-like patterns. Inferior midlateral stripe approximately as wide as orbital diameter. Anal-fin base stripe approximately as wide as orbital diameter. Superior midlateral stripe and inferior midlateral stripe separated by weakly pigmented area, absent in juveniles (Fig 4). Head densely covered by dark brown chromatophores gradually more spaced ventrally. Pectoral and anal fins hyaline with scattered dark brown chromatophores overlying fin rays.

### Color in life

Based on photographs taken in field (Fig 5). Overall body translucent, darker in mid-dorsal region of trunk; dorsal region of body yellowish brown in large specimen (95.9 mm *L*_EA_) (Fig 5A) or greyish yellow in small specimen (79.0 mm *L*_EA_) (Fig 5B). Dorsal region of head dark brown, ventral region lighter, with diffuse chromatophores. Opercle translucent in small specimens, exposing red color of gills; opercle light brown with a concentration of black chromatophores in large specimens. Silvery abdominal region due presence of iridophores covering external portion of peritoneal membrane, more evident in small than large specimens. Midlateral stripes equally located and similar to those found in fixed specimens. Pectoral and anal fins hyaline with scattered dark brown chromatophores overlying fin rays.

## Etymology

The specific epithet *sirius* is an allusion to the alpha star of the Canis Major constellation that represents the state of Mato Grosso in the Brazilian national flag, a reference of the state of occurrence of the new taxon. A noun in apposition.

### Distribution and habitat

**Fig 9:**
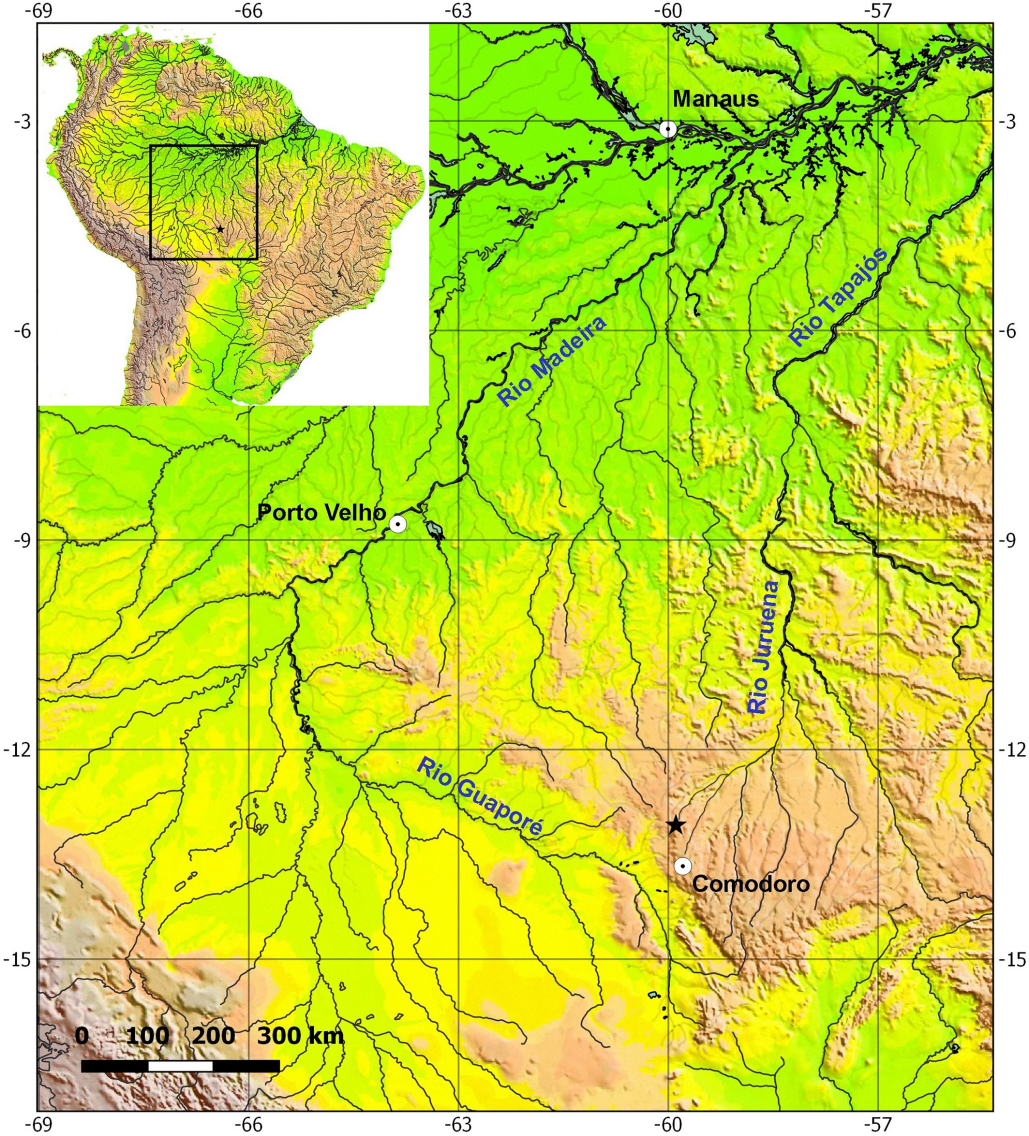
Map of the upper portion of rio Tapajós basin, showing the locality of *Eigenmannia sirius*. Star represents more than one lot.

**Fig 10:**
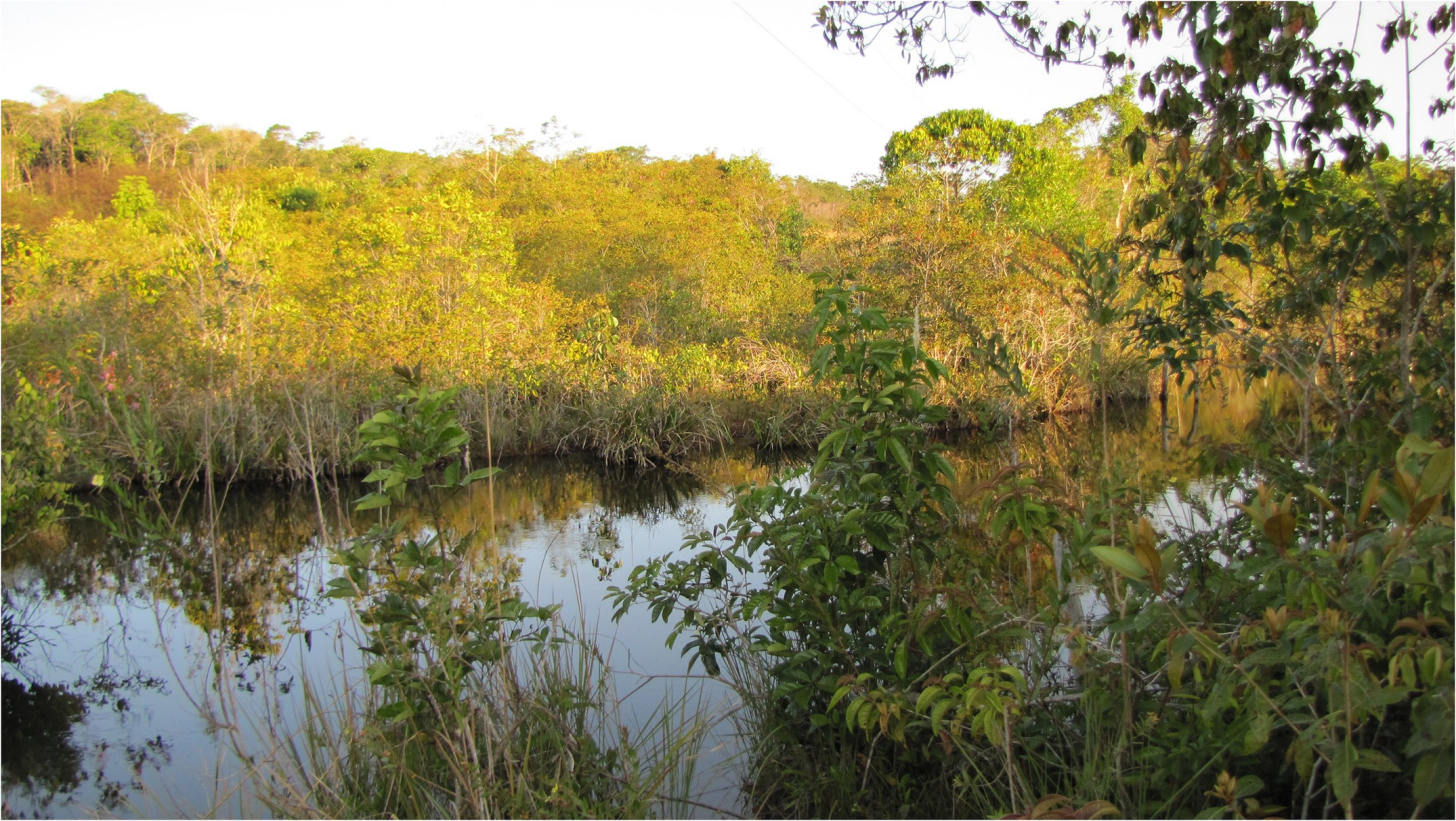
Photograph of rio Mutum, type locality of *Eigenmannia sirius*, illustrating habitat.

*Eigenmannia sirius* is currently known only from rio Mutum, a tributary of the upper rio Juruena, rio Tapajós basin, Comodoro, Mato Grosso, Brazil (Fig 9). The type-locality is 502 m above sea level at the Chapada dos Parecis plateau. It is a clearwater river up to *c*. 3–6 m wide and 0.5–2.5 m deep, preserved riparian vegetation, swift current, and sand, pebbles and dead leaves on the bottom (Fig 10). Several types of microenvironment were sampled exhaustively, but *E*. *sirius* was captured only between root and subaquatic vegetation. Other species sampled syntopically were *Aequidens* cf. *rondoni* (Miranda Ribeiro), *Erythrinus erythrinus* (Bloch & Schneider), *Hemigrammus skolioplatus* Bertaco & Carvalho, *Hyphessobrycon hexastichos* Bertaco & Carvalho, *H*. *melanostichos* Carvalho & Bertaco, *Hasemania nambiquara* Bertaco & Malabarba, and *Hoplerythrinus unitaeniatus* (Spix & Agassiz) (for other species see [46]). No other Gymnotiformes were collected with *E*. *sirius*.

### Conservation status

*Eigenmannia sirius* is currently known only from its type-locality, and it may have a very restricted distribution. However, considering that no imminent threats to the species were detected in the area of its occurrence, *E*. *sirius* would be classified as Least Concern (LC) according to the International Union for Conservation of Nature (IUCN) categories and criteria [47].

## Discussion

### Myological analysis across Sternopygidae

Anatomical studies in Gymnotiformes follow the trend of efforts implemented in other Teleostei groups and focused on detailed descriptions of distinct osteological complexes (e.g., [15,32,48]), neuroanatomical structures or anatomical components associated with electrogenesis and electroreception (e.g., [37,38,49–51]). Recently, efforts were employed aiming to describe structures recently discovered in Gymnotiformes, like the caudal skeleton in *Electrophorus* Gill [52] and pseudotympanum [53]. Thus, studies of comparative anatomy in Gymnotiformes trends to explore traditional sources of information, resulting in a hiatus on several anatomical complexes that can be crucial in understanding the evolution of the group, such as myological tissues, which have rarely been explored [34,54–56]. As a result, characters derived from myology represents less than 0.2% of the entire universe explored of the morphological traits in the cladistic studies in Gymnotiformes (e.g., [18–21,42,57,58]). After a myological analysis, additional data derived from this poorly explored source of data led us to infer putative useful variations in a phylogenetic scenario.

**Fig 11:**
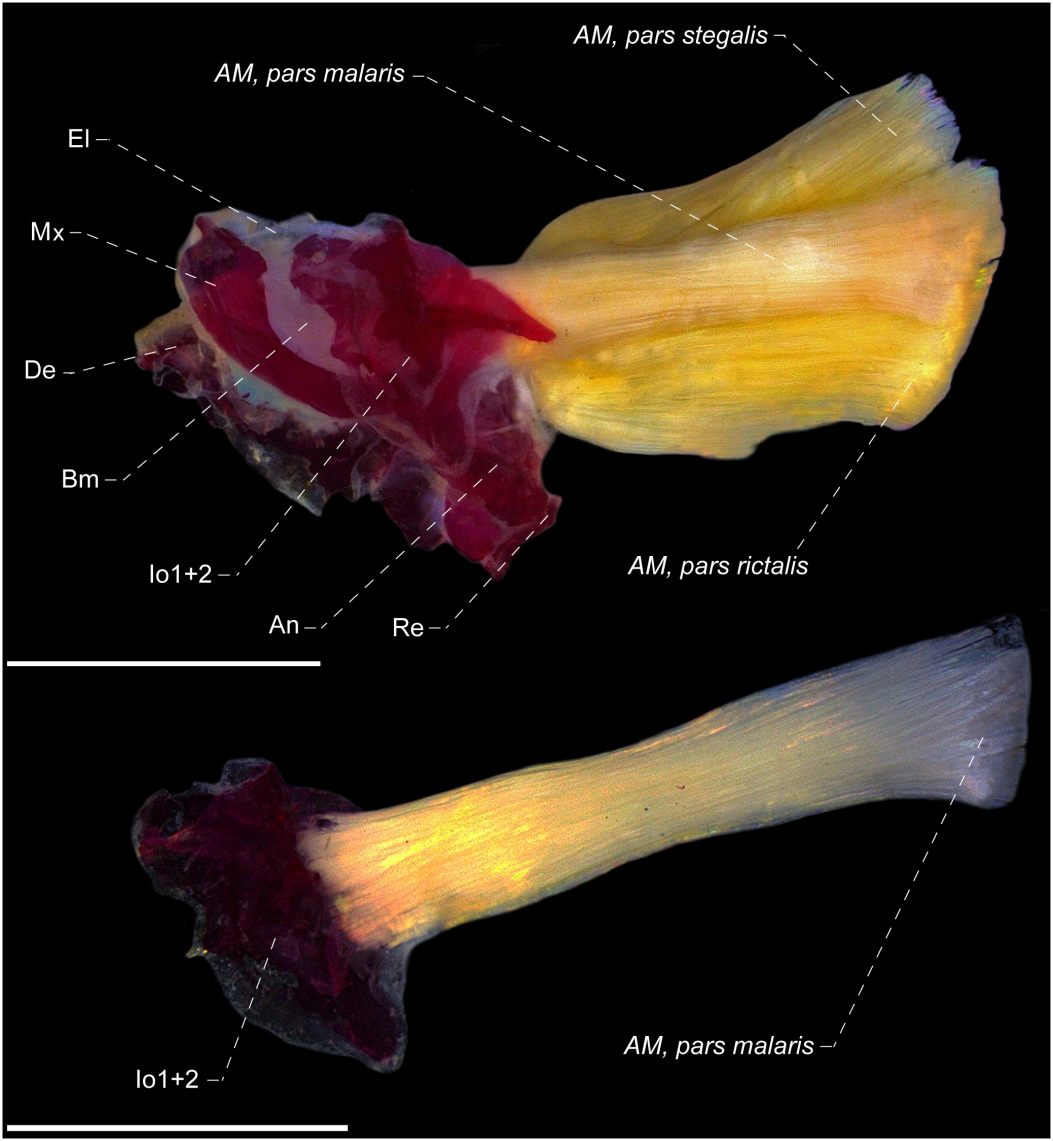
Lateral view of *adductor mandibulae*, *segmentum facialis* (A) and medial view of *adductor mandibulae*, *pars malaris* (B) of *Sternopygus astrabes*, MZUSP 88795, 151.0 mm *L*_EA_. Am = *adductor mandibulae*; An = anguloarticular; Bm = buccopalatal membrane; De = dentary; El = endomaxilar ligament; Io = infraorbital; Mx = maxillary bone; Re = retroarticular. Endomaxilar ligament and buccopalatal membrane dissected. Scale: 3 mm.

**Fig 12:**
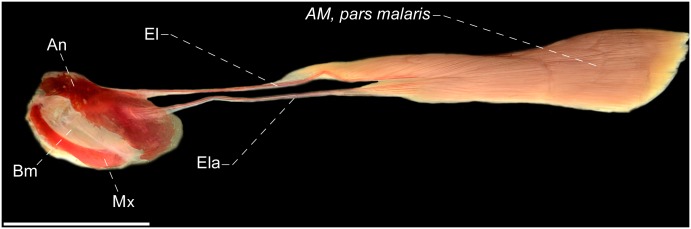
Lateral view of *adductor mandibulae*, *pars malaris* of *Rhamphichthys hahni*, MZUSP 24736, 479.5 mm. Am = *adductor mandibulae*; Ao = antorbital; Bm = buccopalatal membrane; El = endomaxilar ligament; Ela = endomaxilar ligament accessory; Mx = maxillary bone. Scale: 1 cm.

The Sternopygidae genera presents the insertion of the *adductor mandibulae*, *pars malaris* occurring primary in the mesial face of the posterodorsal expansion of the infraorbital 1+2 (Fig 11). Towards its insertion, the mesialmost fibers of the *pars malaris* converge onto an endomaxilar ligament which, in turn, inserts in the mesial face of the posterodorsal portion of the maxillary bone. This configuration is an exclusive condition shared in all Sternopygidae genera, which differs from remaining Gymnotiformes families, and has been proposed as a synapomorphy for Sternopygidae, however, with superficial homology inferences on the sections of the *adductor mandibulae* (see Datovo & Vari, [28,29] for discussions on the homology of *adductor mandibulae* sections). For instance, previous studies [20,22] proposed the aforementioned condition as a homoplastic feature found in *Rhamphichthys* Müller & Troschel, however, in this genus, the *pars malaris* converge onto a wide endomaxilar ligament which, in turn, inserts onto the mesial face of the antorbital and maxillary bones (Fig 12) (per. obs.; [34]), without association with any infraorbital bone, thus non-homologous with those condition found in Sternopygidae genera. Additionality, the ventral fibers of *Rhamphichthys* converge in a ventral ligament, named herein as “accessory endomaxilar ligament”, which inserts only to the mesial face of the antorbital. The monophyly of Sternopygidae was questioned only by studies grounded in molecular data [18,22], with its monophyly well-corroborated in several studies from distinct source of data [15,16,19–21,40–43,49], including the aforementioned myological data source.

**Fig 13:**
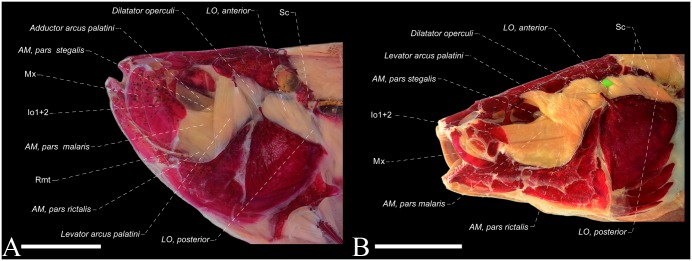
Lateral view of the dorsolateral head muscles of: A) *Rhabdolichops troscheli*, MZUSP 57704, 104.2 mm *L*_EA_ and B) *Sternopygus macrurus*, MPEG 22756, 245.8 mm *L*_EA_. Am = a*dductor mandibulae*; Io = infraorbital; Mx = maxillary bone; Sc = supracleithrum + posttemporal canal; LO = *Levator operculi*; Rmt = *ramus mandibularis trigeminus*. Recurrent ramus of anteroventral part of anterior lateral line nerve in green. Scales: 3 mm (A) and 1 cm (B).

**Fig 14:**
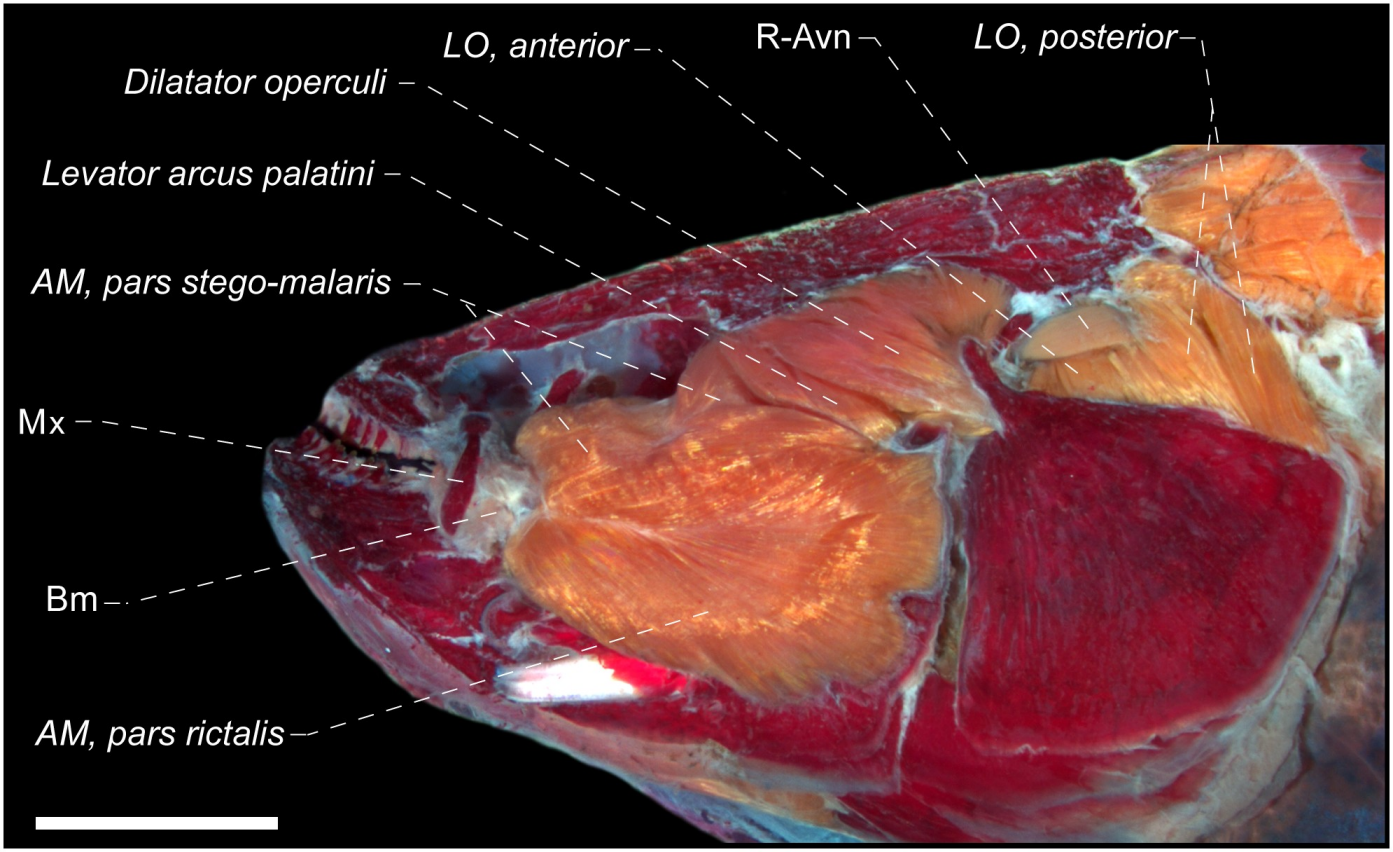
Lateral view of the dorsolateral head muscles of *Gymnotus cylindricus*, USNM 134701, 178.5 mm *L*_EA_. Am = *adductor mandibulae*; Io = infraorbital; LO = *levator operculi*; Mx = maxillary bone; R-avn = recurrent ramus of anteroventral part of anterior lateral line nerve; Rmt = *ramus mandibularis trigeminus*. Posterodorsal portion of LO, posterior accidentally removed. Scale: 4 mm.

All members of Eigenmanniinae exhibit the *adductor mandibulae*, *pars stegalis* originating from the anterior portion of hyomandibula, metapterygoid and quadrate, without attachment to the sphenotic bone. This condition differs that observed in most gymnotiform species, in which the *pars stegalis* originates from the sphenotic and suspensorial bones. In all Eigenmanniinae genera, except for *Distocyclus*, only the mid-posterior and posterior portion of the *adductor arcus palatini* is located medially to the *pars stegalis* (Fig 13A), resulting in a partial overlapping, which is distinct from the condition found in most Gymnotiformes, where the *pars stegalis* completely overlaps the *adductor arcus palatini* (Figs 13B and 14). In *Distocyclus*, the *pars stegalis* is located laterally only in the region near *adductor arcus palatini* insertion, without overlapping with the mid-posterior or posterior portion of the latter. Furthermore, the R-avn is located medially to the entire *levator operculi* (Figs 8 and 13A) in Eigenmanniinae, that contrasts with the condition found in all other Gymnotiformes analyzed herein where the R-Avn is located laterally to the *levator operculi anterior* and medially to the *levator operculi posterior* (Figs 13B and 14). Thus, grounded in the phylogenetic relationships hypothesized by [21], the following conditions are shared by Eigenmanniinae members: (1) absence of an association of *pars stegalis* with the sphenotic bone, (2) R-avn entirely medial to the *levator operculi*, and (3) partial overlapping between *pars stegalis* and *adductor arcus palatini*, with the loss of the overlapping between the last two muscles in *D*. *conirostris* (Eigenmann & Allen 1942).

Within Eigenmanniinae, only *Archolaemus*, *Distocyclus*, *Eigenmannia* and *Rhabdolichops* exhibit the anterior margin of the *levator arcus palatini* insertion nearly straight in relation to the horizontal arm of the preopercle, resulting in an angle near 90° to the longitudinal axis of the head (Figs 8 and 13A). In contrast, the anterolateral margin of *levator arcus palatini* is arranged obliquely in relation to the longitudinal axis of the head, forming an angle near 45° with the longitudinal axis of the head in most gymnotiforms species, including *Sternopygus* and *Japigny* (Figs 13B and 14). Therefore, the condition found in *Archolaemus*, *Distocyclus*, *Eigenmannia* and *Rhabdolichops* could indicate an useful variation for the elucidation of a close relationship among these four genera within Eigenmanniinae, and due its absence in *Japigny*, could provide an additional evidence for the phylogenetically position of *Japigny* as a sister-group of all Eigenmaniinae genera [59], and not related to *Eigenmannia* species (see Remarks).

### Taxonomic considerations

Although there is no agreement about the relationships within Eigenmanniinae, all genera have been demonstrated as monophyletic assemblages, except for *Eigenmannia*. In an effort to define *Eigenmannia*, Mago-Leccia [16] distinguished the genus among the remaining Sternopygidae by the presence of intermuscular bones at precaudal vertebrae 7 to 9 having a high branched structure, and presence of five branchiostegal rays, the two anteriormost narrow and the three remaining triangular in shape. However, despite their occurrence in *E*. *sirius*, the Mago-Leccia’s definition for *Eigenmannia* has not been recoveredin a phylogenetic scenario based on morphologial (e.g., [19,20]), molecular ([22]; in part) or model-based total evidence [21] data. Due to the doubtful status of *Eigenmannia* as a monophyletic genus, the inclusion of the *E*. *sirius* in *Eigenmannia* is provisionally justified by the presence in the former of the synapomorphies of Eigenmanniinae [15,19–21], and by the absence of those synapomorphies of the remaining Eigenmanniinae genera as discussed by previous studies [9,10,23,25,45]. In addition to our conservative position regarding the proposal of a new genus, we detail the differences between the species described herein and the current definition of all eigenmanniin genera (for external features see Diagnosis).

The new species does not fit in *Japigny* by the absence of single row teeth at the base of the upper oral valve (*vs*. presence); the presence of the contact of the lateral process of the second vertebrae with the parapophysis of the fourth vertebrae (*vs*. absence); and the retroarticular is included in the socket of the lower jaw with the quadrate (*vs*. retroarticular not included in the socket of the lower jaw with the quadrate) [59]. *Eigenmannia sirius* does not fit in the *Archolaemus* definition by the teeth of the first row immobile and firmly attached on the ventral surface of the premaxilla margins (*vs*. anterobasal margins of the teeth of the first tooth row attached to the dentigerous surface of the premaxilla) [59]. *Eigenmannia sirius* can be distinguished from the *Distocyclus* by the presence of endopterygoid teeth (*vs*. absence); dentary with 15–33 teeth arranged in two or three complete rows (*vs*. a single tooth row limited to the anterior portion of dentary) [7]. Further, the new species is distinguished from *Rhabdolichops* by the premaxilla approximately rectangular (*vs*. premaxilla trapezoidal and elongate); one prootic foramen (*vs*. two); the extrascapular independent from the neurocranium (*vs*. extrascapular fused with the neurocranium); and gill rakers short and not ossified (*vs*. gill rakers long and bony) [20,31].

The historical status of *Eigenmannia* as a non-monophyletic genus resulted in a classification based on species groups. The first author who advocates the classification grounded in species group was Alves-Gomes [22], which proposed the clade “*Eigenmannia virescens* species-group” composed by five undescribed species (*Eigenmannia* cf. *virescens* 1, 2, 3, 4 and 5; Alves-Gomes [22]). Few years later, Albert [20] did also not recover the *Eigenmannia* monophyly and proposed its classification in two species group: “*Eigenmannia microstoma* species-group” and “*Eigenmannia virescens* species-group”. In the same study, Albert [20] listed “*Eigenmannia* gr. *macrops*”, composed by *E*. *macrops* and an undescribed species (designated as “*Eigenmannia* sp. B”), however, without discussion about its relationships or classification.

According to Albert [20], the “*Eigenmannia microstoma* species-group” (proposed to allocate *E*. *humboldtii*, *E*. *limbata*, *E*. *microstoma*, *E*. *nigra* and an undescribed species from rio Paraiba) can be distinguished by the body depth in mature specimens with more than 11% *T*_L_ and total length over 350 mm in sexually mature individuals. The *Eigenmannia virescens* species-group *sensu* [20] (composed by *E*. *virescens*, *E*. *trilineata* and an undescribed species from Río Salí, Argentina) was proposed to distinguish species that share the presence of two or three longitudinal stripes on flanks, lateral valvula of cerebellum large, and anterior intermuscular bones highly branched. Despite the valuable contribution to the classification in Gymnotiformes in the early 2000’s, the classification of *Eigenmannia* presented in [20] failed to establish reliable data. Exemplifying, measurement taken as a proportion of total length in Gymnotiformes is not truthful given that fishes often suffer damages in caudal filament by predations, and posteriorly regeneration [60]. Additionally, even when considering only undamaged specimens, the body depth taken as proportion of *L*_EA_ are useful only in interspecific levels within *Eigenmannia*, being irrelevant to supra-specific taxa. Further, *E*. *microstoma* shows four dark longitudinal stripes in flanks [8,61] and anterior intermuscular bones highly branched (MCP 45216). The presence of stripes on flanks in *E*. *microstoma* is mentioned by Albert [20], however, the author did not explain the positioning of *E*. *microstoma* in its group, since the presence of stripes on flanks and the anterior intermuscular bones highly branched are synapomorphies for the *Eigenmannia virescens* species-group. In addition, *E*. *virescens* is characterized by a uniform color pattern, without midlateral stripes (see Peixoto *et al*. [8] for taxonomic discussion of *E*. *virescens*). Peixoto *et al*. [8] did not follow the classification proposed by Albert [20] because of these difficulties in recognizing the characters or the taxonomic compositions and proposed the so called “*Eigenmannia trilineata* species-group”, in which all species share the superior midlateral stripe.

In a recent contribution, Waltz & Albert [10] arbitrarily assigned *E*. *meeki* to the *Eigenmannia trilineata* species-group ([10]: 399; and Table 2 explicitly based on other contributions e.g., [8,23–25,45]). However, the color pattern of *E*. *meeki* is characterized by the lateral line stripe, inferior midlateral stripe and anal-fin base stripe, without the superior midlateral stripe [45], thus, lacking evidences for this proposition.

**Table 2:** Taxonomic arrangement of species group in *Eigenmannia*.

***Eigenmannia humboldtii* species-group**
*Eigenmannia humboldtii* (Steindachner, 1878)
*Eigenmannia limbata* (Schreiner & Miranda-Ribeiro, 1903)
*Eigenmannia nigra* Mago-Leccia, 1994
***Eigenmannia trilineata* species-group**
*Eigenmannia antonioi* Peixoto, Dutra & Wosiacki, 2015
*Eigenmannia besouro* Peixoto & Wosiacki, 2016
*Eigenmannia correntes* Campos-da-Paz & Queiroz, 2017
*Eigenmannia desantanai* Peixoto, Dutra & Wosiacki, 2015
*Eigenmannia guairaca* Peixoto, Dutra & Wosiacki, 2015
*Eigenmannia loretana* Waltz & Albert, 2018
*Eigenmannia matintapereira* Peixoto, Dutra & Wosiacki, 2015
*Eigenmannia microstoma* (Reinhardt, 1852)
*Eigenmannia muirapinima* Peixoto, Dutra & Wosiacki, 2015
*Eigenmannia pavulagem* Peixoto, Dutra & Wosiacki, 2015
*Eigenmannia sayona* Peixoto & Waltz, 2017
*Eigenmannia sirius* Peixoto & Ohara, present study
*Eigenmannia trilineata* López & Castello, 1966
*Eigenmannia vicentespelaea* Triques, 1996
*Eigenmannia waiwai* Peixoto, Dutra & Wosiacki, 2015
**Species not included in any group**
*Eigenmannia macrops* (Boulenger, 1897)
*Eigenmannia meeki* Dutra, de Santana & Wosiacki, 2017
*Eigenmannia oradens* Dutra, Peixoto, de Santana & Wosiacki, 2017
*Eigenmannia virescens* (Valenciennes, 1836)
***Incertae sedis***
“*Eigenmannia*” *goajira* Shultz, 1949

Moreover, Waltz & Albert [26] classified *Eigenmannia* into two groups, besides the *E*. *trilineata* species-group. The so called “*Eigenmannia humboldtii* species-group” *sensu* [26] was proposed as grouping species with: (1) larger body size (>45 cm *T*_L_); (2) deep body at maturity (body depth >11% *T*_L_); and a (3) darker body coloration in some specimens. Subsequently, Waltz & Albert [10] redefined the *Eigenmannia humboldtii* species-group by: (1) large adult body sizes—(>300 mm total length), (2) deep body shape—body depth greater than 18% of the length to the end of anal fin (*L*_EA_), (3) broad and opaque body in life, and (4) absence of longitudinal stripes. In the latter contribution, the authors indicated *E*. *humboldtii*, *E*. *limbata* and *E*. *nigra* as members of the *Eigenmannia humboldtii* species-group. Herein, we corroborate the taxonomic composition of the *Eigenmannia humboldtii* species-group. However, through the discrepancies in the definition of this group, and in addition to the problematic reference to the measurements taken as proportion of *T*_L_ in Gymnotiformes, we redefine the *Eigenmannia humboldtii* species-group to accomplish species that share: (1) the presence of anal fin margin distinctly darkened and (2) absence of longitudinal stripes.

The second group proposed by Waltz & Albert [26] was the“*Eigenmannia macrops* species-group”, including only *E*. *macrops*, and according to authors, distinguished by: (1) body fairly laterally compressed, (2) translucent white/yellow coloration in life, (3) longitudinal stripes absent, (4) eye large (greater than ore qual to snout length), (5) long caudal filament (half of body length without head). Posteriorly, Waltz & Albert [10] exclude the character “body fairly laterally compressed” of its definition. Previously, the “*Eigenmannia macrops* species-group” had already been proposed by [20], and, not only it is composed by only one species, therefore lacking evidence for the proposition of a group of species, but the characters described are generalized features of the remaining *Eigenmannia* species, except for “eye large (greater than or equal to snout length)” and “(5) long caudal filament (half of body length without head)” which are putative diagnostic for *E*. *macrops*. Therefore, there is no evidence for the recognition of a species group designated as *E*. *macrops* until the detection of additional species putatively related to this species.

Consequently, the best resolution is a classification of *Eigenmannia* into two species groups. The striped species compose the *E*. *trilineata* species-group, in which all species share the superior midlateral stripe, and the species with anal-fin margin darkened and without longitudinal stripes on flanks constitute the *Eigenmannia humboldtii* species-group. In addition, we suggest the remaining species on hold for a formal taxonomic review or for having their phylogenetic relationships clarified (e.g. “*Eigenmannia*” *goajira* and *E*. *meeki*; see Table 2). Aiming the summarization of the taxonomic considerations synthetized herein, the first dichotomous key is provided for all *Eigenmannia* species (“*Eigenmannia*” *goajira* not included).

## Remarks

Tagliacollo *et al*. [21] recovered a paraphyletic *Eigenmannia*, with *Japigny* internested within *Eigenmannia* species. Waltz & Albert [26] used the name “*Eigenmannia kirshbaum*” in reference to *Japigny kirshbaum* Meunier, Jégu & Keith, citing the findings of [21] as grounds for the synonimization. However, Tagliacollo *et al*. [21], or even Waltz & Albert [26], did not provide a formal synonym. In addition, Waltz & Albert [10] utilized *Japigny* as a valid name, thus, the recommendation provided herein comprises the utilization of *Japigny* as a valid generic name.

### Key for *Eigenmannia* species (for specimens between 49.4 to 330.0 mm *L*_EA_)

**1**. Longitudinal dark stripes on flanks absent………………………………………………**2**

**1’**. Longitudinal dark stripes on flanks present………………………………………………**6**

**2**. Distinctly darkened anal-fin margin (*Eigenmannia humboldtii* species-group)…………**3**

**2’**. Hyaline anal-fin margin…………………………………………………………………**5**

**3**. Dentary with 53–66 teeth arranged in three rows………………***Eigenmannia humboldtii*** (restricted to the trans-Andean Río Atrato and Río Magdalena, northwestern Colombia).

**3’**. Dentary with 8–37 teeth arranged in one to three rows…………………………………**4**

**4**. Dentary with 8–18 teeth arranged in a single row; dorsal profile of head straight to concave………………………………………………………………***Eigenmannia limbata*** (widespread in Amazon and Orinoco basins).

**4’**. Dentary with 27–37 teeth arranged in two or three rows; dorsal profile of head strongly convex……………………………………………………………………***Eigenmannia nigra*** (widespread in Amazons, Araguaia-Tocantins, Essequibo and Orinoco basins).

**5**. Orbital diameter, 15.6–24.8% *H*_L_; caudal filament length, 17.1–31.1% *L*_EA_……………………………………………………………………………………***Eigenmannia virescens*** (lower Paraná and de La Plata basins).

**5’**. Orbital diameter, 28.0–32.8% *H*_L_; caudal filament lenght, 40.2–52.7% *L*_EA_………………………………………………………………………………….***Eigenmannia macrops*** (Potaro, Orinoco and Essequibo basins).

**6**. Superior midlateral dark stripe on flanks absent…………………………………………**7**

**6’**. Superior midlateral dark stripe on flanks present (*Eigenmannia trilineata* species-group)………………………………………………………………………………………**8**

**7**. Bony dorsolateral flange of dentary present which anchors numerous teeth along its extension; 38–42 teeth on premaxilla; 164–192 anal-fin rays…………***Eigenmannia oradens*** (Río Ventuari, Río Orinoco basin).

**7’**. Bony dorsolateral flange of dentary absent; 30–35 teeth on premaxilla; 211–240 anal-fin rays……………………………………………………………….***Eigenmannia meeki*** (Río Pucuro and Río Chucunaque, Río Tuíra basin).

**8**. Pectoral fin dusky or with conspicuous dark blotch; anal fin uniformly darkened………………………………………………………………………… ***Eigenmannia matintapereira*** (rio Uneiuxi and rio Urubaxi, rio Negro basin).

**8’**. Pectoral and anal fins hyaline………………………………………………………….**9**

**9**. Mouth subterminal…………………………………………………………………….**10**

**9’**. Mouth terminal……………………………………………………………………….**14**

**10**. Body depth at vertical through the tip of the longest pectoral-fin ray, 10.5–14.5% *L*_EA_; seven or eight longitudinal series of scales above lateral line; length of the coronomeckelian bone equal to 45% of the length of Meckel’s cartilage………………………………………………………………………………. ***Eigenmannia vicentespelaea*** (caves of São Vicente I and II, rio Tocantins basin).

**10’**. Body depth at vertical through the tip of the longest pectoral-fin ray, 12–18.8% *L*_EA_; seven to 12 longitudinal series of scales above lateral line; length of the coronomeckelian bone equal to 20–30% of the length of Meckel’s cartilage………………………………**11**

**11**. Origin of the superior midlateral stripe, at vertical between base of 5^th^ to 15^th^ anal-fin ray in adults……………………………………………………………***Eigenmannia besouro*** (rio São Francisco basin).

**11’**. Origin of the superior midlateral stripe, at vertical between base of 23^th^ to 32^th^ anal-fin ray in adults……………………………………………………………………………… **12**

**12**. Precaudal vertebrae, 12–13; 35–40 teeth on premaxillary; 37–38 teeth on dentary; orbital diameter, 22.6–28.8% *H*_L_; nine or ten longitudinal series of scales above lateral line…………………………………………………………………. ***Eigenmannia waiwai*** (rio Mapuera and rio Trombetas, rio Trombetas basin).

**12’**. Precaudal vertebrae, 14–15; 15–24 on premaxillary teeth; 16–25 teeth on dentary; orbital diameter, 10.0–23.3% *H*_L_; nine to 12 longitudinal series of scales above lateral line…………………………………………………………………………………………**13**

**13**. Relative depth of posterodorsal expansion on infraorbitals 1+2 corresponding to 70% length of infraorbitals 1+2; precaudal vertebrae, 15; orbital diameter, 17.2–23.8% *H*_L_; mouth width, 13.1–22.4% *H*_L_……………………………………. ***Eigenmannia sirius* n.sp**. (Rio Mutum, a tributary of rio Juruena, rio Tapajós basin).

**13’**. Relative depth of posterodorsal expansion on infraorbitals 1+2 corresponding to 40% length of infraorbitals 1+2; precaudal vertebrae, 14; orbital diameter, 10.6–13.3% *H*_L_; mouth width, 23.5–26.0% *H*_L_……………………………………. ***Eigenmannia correntes*** (rio Correntes, a tributary of rio Piquiri, rio Paraguai basin).

**14**. Suborbital depth, 28.3–46.6% *H*_L_………………………………………………………**15**

**14’**. Suborbital depth, 18.2–28.9% *H*_L_…………………………………………………….**18**

**15**. Relative depth of posterodorsal expansion on infraorbitals 1+2 corresponding to 60–100% length of infraorbitals 1+2; 11–16 teeth on premaxillary; 16–19 teeth on dentary……………………………………………………………………………………**16**

**15’**. Relative depth of posterodorsal expansion on infraorbitals 1+2 corresponding to 50% length of infraorbitals 1+2; 31–33 teeth on premaxillary; 31 teeth on dentary………………………………………………………………………………………***Eigenmannia trilineata*** (lower rio Paraná and Río de La Plata basins).

**16**. Length of coronomeckelian bone 45% length of Meckel’s cartilage; 11–16 arranged in one or two rows on endopterygoid……………………………***Eigemannia microstoma*** (rio São Francisco basin).

**16’**. Length of coronomeckelian bone 20–30% length of Meckel’s cartilage; 6–9 teeth arranged in one row on endopterygoid…………………………………………………….**17**

**17**. First basibranchial ossified; suborbital depth, 20.6–26.8% *H*_L_……***Eigemannia sayona*** (río Orinoco basin).

**17’**. First basibranchial unossified; suborbital depth, 28.3–35.8% *H*_L_…………………….………………………………………………………………………***Eigemannia loretana*** (río Pacaya and río Nanay basins).

**18**. Inferior midlateral stripe, one scale high; precaudal vertebrae, 11–12…………………………………………………………………………………………***Eigenmannia desantanai*** (rio Cuiabá, rio Paraguai basin).

**18’**. Inferior midlateral stripe, two or three scale high; precaudal vertebrae, 13–15………**19**

**19**. Pectoral-fin rays, ii,11–12………………………………………………………………**20**

**19’**. Pectoral-fin rays, ii,13–15……………………………………………………………**21**

**20**. Orbital diameter, 15.4–19.4% *H*_L_; 170–198 anal-fin rays; eight or nine endopterygoid teeth; 13–14 precaudal vertebrae…………………………………***Eigenmannia muirapinima*** (Igarapé Santo Antônio and Lago Jará, rio Amazonas basin).

**20’**. Orbital diameter, 11.4–15.0% *H*_L_; 151–170 anal-fin rays; five or six endopterygoid teeth; 15 precaudal vertebrae………………………………………***Eigenmannia guairaca*** (upper rio Paraná basin).

**21**. Width mouth, 20.0–25.1% *H*_L_; 11–13 teeth on premaxillary……***Eigenmannia antonioi*** (rio Anapu, rio Amazonas).

**21’**. Width mouth, 10.8–19.0% *H*_L_; 15–21 teeth on premaxillary………………………………………………………***Eigenmannia pavulagem*** (Rio Capim, rio Guamá basin).
